# Comprehensive Study of Oxygen Vacancies on the Catalytic Performance of ZnO for CO/H_2_ Activation Using Machine Learning-Accelerated First-Principles Simulations

**DOI:** 10.1021/acscatal.3c00658

**Published:** 2023-03-30

**Authors:** Yulan Han, Jiayan Xu, Wenbo Xie, Zhuozheng Wang, P. Hu

**Affiliations:** † School of Chemistry and Chemical Engineering, Queen’s University Belfast, Belfast BT9 5AG, UK; ‡ PetroChina Petrochemical Research Institute, Beijing 102206, China; § School of Physical Science and Technology, ShanghaiTech University, 393 Middle Huaxia Road, Shanghai 201210, China

**Keywords:** oxygen vacancy, syngas, machine learning potential, genetic algorithm, DFT

## Abstract

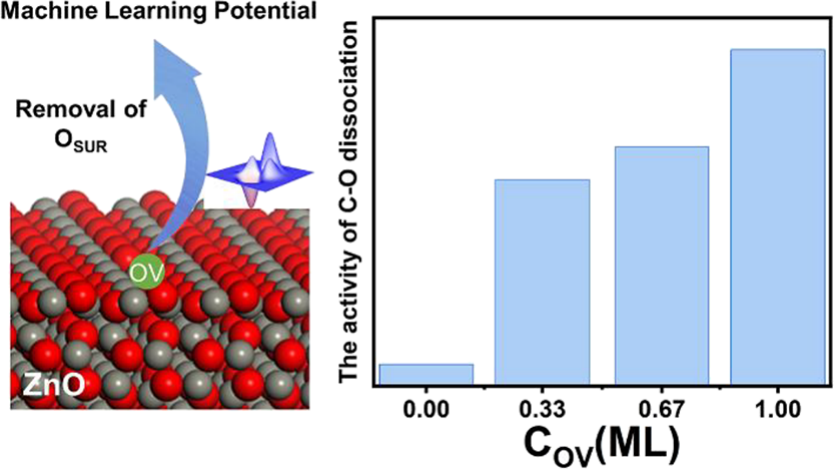

Oxygen vacancies (OVs) play important roles on any oxide catalysts. In this work, using an investigation of the OV effects on ZnO(101̅0) for CO and H_2_ activation as an example, we demonstrate, via machine learning potentials (MLPs), genetic algorithm (GA)-based global optimization, and density functional theory (DFT) validations, that the ZnO(101̅0) surface with 0.33 ML OVs is the most likely surface configuration under experimental conditions (673 K and 2.5 MPa syngas (H_2_:CO = 1.5)). It is found that a surface reconstruction from the wurtzite structure to a body-centered-tetragonal one would occur in the presence of OVs. We show that the OVs create a Zn_3_ cluster site, allowing H_2_ homolysis and C–O bond cleavage to occur. Furthermore, the activity of intrinsic sites (Zn_3c_ and O_3c_ sites) is almost invariable, while the activity of the generated OV sites is strongly dependent on the concentration of the OVs. It is also found that OV distributions on the surface can considerably affect the reactions; the barrier of C–O bond dissociation is significantly reduced when the OVs are aligned along the [12̅10] direction. These findings may be general in the systems with metal oxides in heterogeneous catalysis and may have significant impacts on the field of catalyst design by regulating the concentration and distribution of the OVs.

## 1 Introduction

Syngas (CO/H_2_) is an important intermediate platform for the utilization of carbon resources such as coal, natural gas, and biomass, which can be converted into a variety of high-value chemicals and fuels.^1,2^ Great efforts have been devoted to understanding the reaction mechanisms of syngas conversion. It is generally believed that methanol synthesis through CO hydrogenation catalyzed by Cu/ZnO/Al_2_O_3_ follows a sequential hydrogenation mechanism to formyl and methoxy intermediates and eventually methanol.^3^ However, for the syngas-to-olefin reaction, syngas is proposed to be converted to ketene over the ZnCrO_*x*_ catalyst,^4^ while CH_3_OH is converted over the Zr–Zn catalyst.^5^ ZnO-based catalysts, such as Zn_*x*_Cr_*y*_O_*z*_, Zn_*x*_Zr_*y*_O_*z*_, Zn_*x*_In_*y*_O_*z*_, Zn_*x*_Al_*y*_O_*z*_, and Zn_*x*_Ga_*y*_O_*z*_, have been widely used for converting syngas into methanol and have recently gained much attention as a key ingredient in bifunctional oxide catalysts for syngas to light olefin (STO) conversion.^6−13^ Incorporation of ZnO into other metal oxides can enhance the H_2_ dissociation reactivity, thus accelerating the hydrogenation process. However, the overly strong hydrogenation ability is the major reason why conventional methanol synthesis catalysts such as Zn_*x*_Al_*y*_O_*z*_ are not suitable as the active component for the STO process. Thus, moderate regulation of the H_2_ dissociation and hydrogenation ability is important for obtaining target products.

In addition to these two reactions, the energy barrier (*E*_a_) of C–O dissociation has an important effect on the selectivity of the intermediates (CH_3_OH vs CH_2_CO), which enter the zeolite for further reaction. The easier it is to break the bond, the more likely it is to form CH_2_CO.^14^ As such, the most important step in understanding the complex behaviors of syngas conversion over ZnO-involved catalysts is the H_2_/CO activation.

Zinc oxide is well known to be an oxygen-deficient material. It was discovered that the presence of oxygen vacancies (OVs), i.e., the concentration and the distribution of OVs, determined the catalytic activity of metal oxides. For instance, Wei et al. have reported that the OVs on the ZnO(101̅0) surface promoted the dissociative adsorption of O_2_.^15−17^ Polarz et al. have investigated the hydrogenative conversion of CO to methanol over ZnO nanostructures containing different amounts of OVs.^18^ In addition, Xiao et al. have shown that there is an evolution of product selectivity as the concentration of OVs on ZnO(0001̅) increases under experimental conditions with different H_2_/CO ratios.^19^ This naturally raised some questions: (i) How does the surface structure evolve under different chemical potentials of oxygen related to the H_2_/CO pressures and experimental temperatures? (ii) How do OVs (concentration and distribution) affect the activation of H_2_ and CO? (iii) Do these OVs directly serve as the active sites, or do they indirectly affect the activities of intrinsic sites?

To understand the relationship between the structure and activity of catalysts, the identification of the real surface structure is of great importance. The catalyst morphology can evolve under different reaction conditions, and many heterogeneous catalyst structures are disordered or amorphous in their active state, which complicates the identification of the active site and structure–property relations. However, most theoretical structure searching is based on density functional theory (DFT) calculations, which are computationally expensive and confined to limited databases, leading to the systems studied being very small. Recent advances in machine learning (ML) approaches to construct high-dimensional machine learning potentials (MLPs) have shown great potential in balancing between accuracy and cost, achieving the accuracy close to that of DFT with the cost of empirical potentials.^20^ Many groups are devoted to employing ML to accelerate global optimization methods—for example, the stochastic surface walking (SSW),^21^ genetic algorithm (GA)-based methods^22^ and particle swarm optimization (PSO) methods^23^—revealing some intriguing findings of the structure searches. To date, several MLPs have been successfully developed, such as the large-scale atomic simulation with neural network potential (LASP),^24^ deep-neural network potential (DNNP),^25^ embedded-atom neural network potential (EANNP),^26^ and Gaussian approximation potential (GAP).^27^ Nevertheless, the construction of MLPs requires a large and representative potential energy surface (PES) dataset computed a priori by DFT calculations. The creation of high-quality training sets involving the generation and selection of available data is of great importance to the accuracy of the PES.

In this work, we developed an active learning scheme to accelerate the construction of MLPs of ZnO to address the questions mentioned above. Using the GA-based global structural optimization method accelerated by MPLs, we successfully identified the most likely ZnO(101̅0) surface with various concentrations of OVs after searching over hundreds of thousands of configurations. In the presence of OVs, a wurtzite (WZ)-to-body-centered-tetragonal (BCT) surface reconstruction can be seen. ZnO(101̅0) with 0.33 ML OVs is found to be the most stable structure by evaluating the thermodynamic stability under the reaction conditions. With the increase in the OV concentration, the activity of the intrinsic sites (Zn_3c_ and O_3c_ sites) is hardly altered, whereas that of the new OV site increases gradually due to the localization of the extra electrons and structure deformations derived from the departure of oxygen. Perhaps more importantly, we find that the C–O bond dissociation barrier is significantly reduced when the OVs are distributed along the [12̅10] direction, thus affecting the product selectivity. The obtained results could provide new insights into the role of oxygen vacancies in CO and H_2_ activation at the molecular level.

## 2 Methods

### 2.1 DFT Calculations

In this work, all spin-polarized DFT calculations were performed using the Vienna Ab Initio Simulation Package (VASP)^28,29^ with projector-augmented-wave (PAW) pseudopotentials for the plane-wave basis expansion (PBE). The cut-off energy is 400 eV. The density of states (DOS) of the critical structures was calculated using the hybrid HSE06 functional in order to obtain an accurate description of the electronic structure.^30^

Owing to the high computational cost of HSE06, the PBE + U was used to optimize the structures because of the good compromise between the accuracy and computational cost.^31−33^ The U value was empirically set of 4.0 eV for Zn 3d orbitals.^34^ Unless stated otherwise, all energetics data reported in this work are those obtained from the PBE + U functional. The slab was separated by a vacuum of 15 Å in the *z*-direction to ensure negligible interaction between the slab and its periodic images. Constrained minimization was used to search transition states (TSs) that were further verified by vibrational frequencies.^35−39^ The adsorption energy (*E*_ads_) of an adsorbate on the ZnO surface was calculated as the energy difference between the adsorbate–ZnO complex and the sum of isolated ZnO and the adsorbate. The reported binding energies correspond to the energetically most favorable configurations. Note that negative binding energies signify attractive interactions.

### 2.2 Genetic Algorithm Optimization Methods

**Figure 1:**
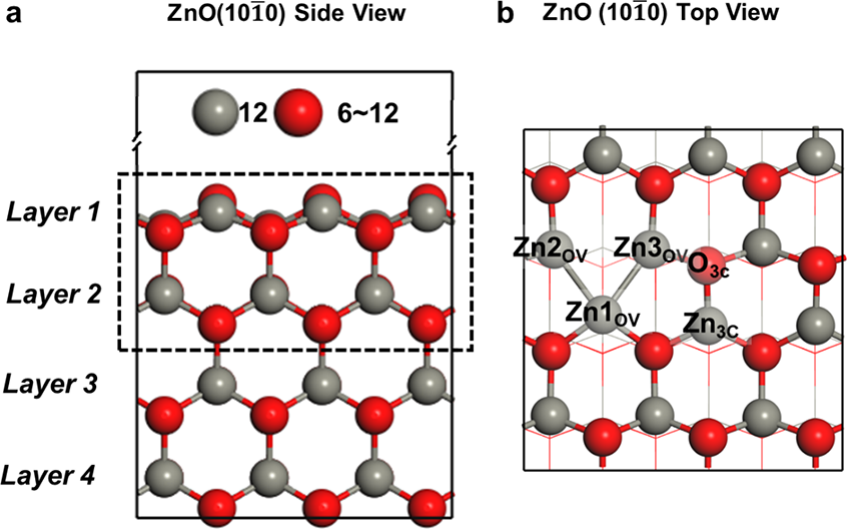
Side view and top view of the ZnO(101̅0) model are illustrated in (a) and (b), respectively. The GA explored the configurations of ZnO_*x*_ (*x* = 6–12) with the top two atomic layers shown in the dashed rectangle. The third layer can be relaxed while the fourth layer is frozen during the optimization process. Zn*n*_OV_ (*n* = 1–3) represents the new sites due to the departure of oxygen, while Zn_3c_ and O_3c_ denote intrinsic three-coordination sites on the surface.

Note that the ZnO mainly exposes the (101̅0) facets due to the lowest surface energy.^40−42^ Therefore, we choose the ZnO(101̅0) surface as an example in this work. Figure 1 shows the *p*(3 × 2) ZnO(101̅0) model, and the main sites on the surface are shown in Figure 1b. According to the size of the unit cell, the Brillouin zone was sampled by a (2 × 2 × 1) Γ-centered Monkhorst–Pack mesh. The atoms in the first three layers are relaxed, while the remaining atoms are fixed during the structure optimization.

We used the GA optimization method implemented in the atomic simulation environment (ASE) to search for the most likely structures of ZnO(101̅0) with different concentrations of OVs (Figure 1).^43−45^ The detailed implementation of this methodology is described in the Supporting Information (SI). The parameters of the GA in this study are as follows: initial population size: 100, crossover probability: 1, mutation probability: 0.5 (rattle:mirror:permutation = 1:1:1), generation: 20. We first carried out optimization using the MLPs to explore a large set of structures, while the metastable and global minimum (GM) structures based on the GA optimization are refined using DFT with settings listed above.

The concentration of surface OV is defined as follows:
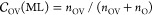
1where *n*_OV_ and *n*_O_ are the numbers of OV and O atoms on the outmost layer (the unit is monolayer (ML)), respectively.

To assess the stability of the ZnO(101̅0) surface with various numbers of OVs, we use the Gibbs free formation energy as
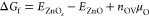
2

As syngas conversion takes place in a reduction atmosphere with a high temperature and pressure, there should be a surface structure evolution under the reaction conditions. The chemical potential of oxygen (μ_O_) was estimated by the experimental conditions.

In case the surface is equilibrated with CO producing CO_2_, μ_O_ is expressed by
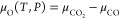
3

Otherwise, the surface is equilibrated with H_2_ producing H_2_O, μ_O_ is expressed by
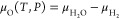
4
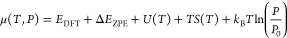
5where *E*_DFT_ can be obtained from the DFT total energy. Vibrational frequency calculations were performed to determine the zero-point energy (Δ*E*_ZPE_). *U*(*T*) is the enthalpy correction and *S*(*T*) is the entropy taken from the NIST database. According to the experimental conditions, we used a temperature of 673 K and a pressure of 2.5 MPa (H_2_/CO = 1.5). More details can be found in the SI.

The Boltzmann distributions based on the Gibbs free energy of formation have been calculated as:^45^
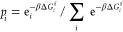
6Where *p_i_* is the probability of a minimum, Δ*G*_i_^f^ is the formation energy between the given structure and the perfect surface, and β is 1/*K*_B_*T*.

### 2.3 Active Learning for Accelerating GA Global Optimization

We used the new embedded atom neural network (EANN)^26^ method to develop an accurate and extremely complex potential energy surface. The EANN approach is equally accurate as several established ML models in representing both large and extended periodic systems yet with much fewer parameters and configurations.^46,47^ It is highly efficient as it implicitly contains the three-body information without an explicit sum of the conventional costly angular descriptors.

**Figure 2:**
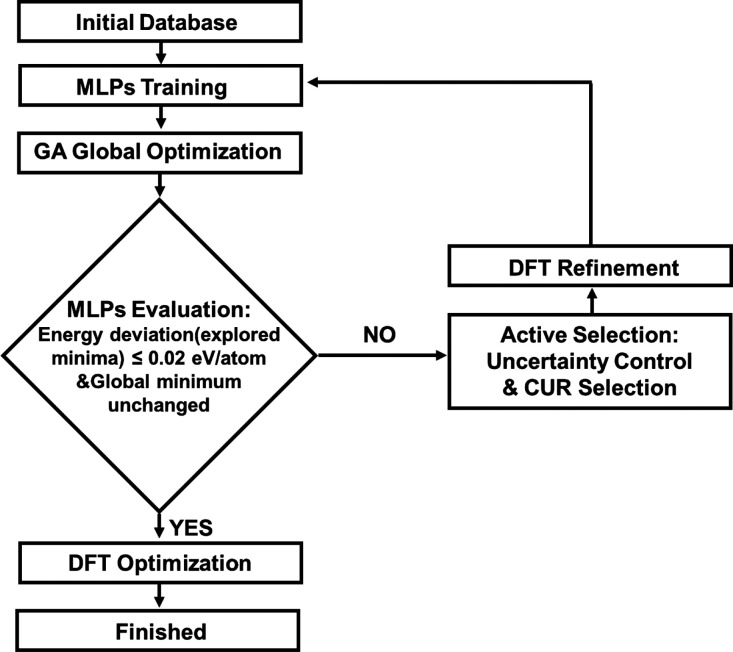
Flow chart of the active learning algorithm for accelerating GA global optimization.

The MLPs were paired with the active learning procedure shown in Figure 2, which involves an iterative process:

(1) building up a training dataset manually using DFT calculations;
(2) training of the MLPs;
(3) sampling the structures using the GA-based global optimization method;
(4) using the committee model strategy to estimate the uncertainty of model predictions and using CUR decomposition to further sift representative structures; and
(5) further performing DFT calculations to augment the dataset.

The detailed implementation of this methodology is described in the SI.

In this work, EANNP uses six Gaussian-type radial functions with automatically learned parameters and third-order (*L* = 3) angular expansion for the atomic representation. A cut-off of 6.0 Å is considered for neighboring atoms. The atomic-energy fitting neural network uses a hidden-layer architecture of 256 × 128 × 64 × 32. The uncertainty criteria for the unlearned configurations are set to be 0.05–0.25 eV/atom for energy. The detailed list of the training dataset, containing 55 764 structures, can be found in Table S1. As shown in Figure S1, the overall performance of EANNP on the complete dataset reaches a root mean square error (RMSE) in energy of 0.036 eV/atom, and a RMSE in forces of 0.192 eV/Å for O and 0.167 eV/Å for Zn.

## 3 Results

### 3.1 Stability of the ZnO Surfaces

**Figure 3:**
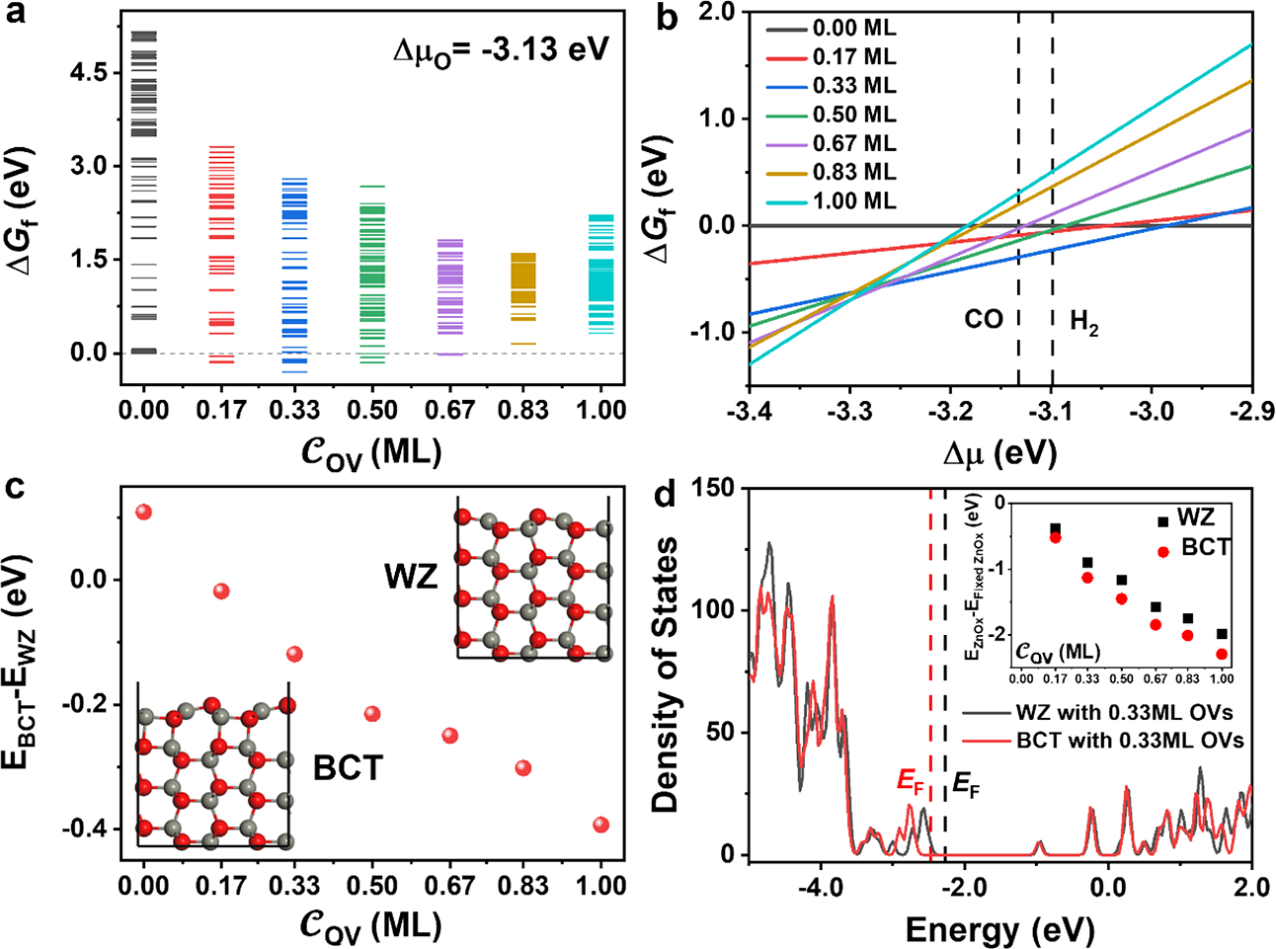
(a) Thermodynamic convex hull for ZnO(101̅0) with different OV concentrations referring to the perfect ZnO(101̅0) surface under the experimental condition (Δμ_O_= −3.13 eV). The chemical potential of surface oxygen is referenced to a gaseous O_2_ molecule. (b) Phase diagram made up of global minima (GM) for ZnO (101̅0) with different OV concentrations at 673 K and a pressure of 2.5 MPa. In the H_2_-rich zone, the ZnO surface is reduced by H_2_, while in the CO-rich zone, the ZnO surface is reduced by CO. (c) Energy difference between the BCT surface and WZ surface as a function of OV concentration. The inset shows the side views of the BCT and WZ surfaces, respectively. (d) Density of states (DOS) of the WZ and BCT surfaces with 0.33 ML OVs, and the inset is the computed energy difference between the fixed and relaxed configurations as a function of OV concentration.

In order to determine the most thermodynamically stable configurations of ZnO surfaces with various concentrations of OVs, the GA method with the trained PES was used to explore the configurational space, and further DFT calculations were performed on the top 100 stable configurations obtained by the trained PES. In this work, seven different concentrations of the surfaces were studied. Hundreds of thousands of structures per composition are explored using MLPs, which provides a foundation for the GM structural determination. For all the ZnO(101̅0) configurations with various concentrations of OVs, the DFT-generated convex shell and the phase diagram with critical structures we discovered are shown in Figure 3. Figure 3a illustrates the convex shell formed by our 700 explored configurations, derived from the top 100 most stable structures validated by DFT calculations at each OV concentration, of which any defect phases with negative formation energies are favorable to form from the primary stoichiometric surface. We find that the ZnO(101̅0) surface with 0.33 ML OVs is the most stable phase with a formation energy of −0.30 eV under the experimental condition. The structure ensemble under the reaction conditions consists of four structures with negative formation energies, namely, GM for ZnO(101̅0) surface with 0.17, 0.33, 0.50, and 0.67 ML OVs, the Boltzmann populations of which are 0.28, 99.40, 0.32, and 0.00%, respectively. In addition, unit cells with different sizes and symmetries were also explored to further prove that the ZnO(101̅0) surface with 0.33 ML OVs is the most likely configuration under the experimental conditions (Figures S2−S3).

Then, we investigate the stability trend of the GM surfaces for each composition over a range of oxygen chemical potentials. As shown in Figure 3b, four surfaces, i.e., stoichiometric surface, surfaces with the OVs of 0.33, 0.67, and 1.00 ML, are found to be stable under different chemical potentials of oxygen. Under the relatively O-rich conditions, the stoichiometric surface is the most stable surface, which is reasonable. Under the experimental conditions, ZnO with 0.33 ML OVs is more stable than other surfaces in a H_2_-rich or CO-rich atmosphere. As the chemical potential of oxygen decreases, ZnO with the OVs of 0.67 ML and then 1.00 ML become more stable.

An interesting result is discovered, as illustrated in Figure 3c; namely, the outermost atomic layers of the ZnO(101̅0) surfaces can be reconstructed from the WZ to BCT lattice when OVs are created on the surfaces. With the increasing concentration of OVs, the energy difference between the BCT and WZ surface structures of the same composition becomes larger. The same phenomenon can be found for other surfaces of various symmetries and sizes in Figure S4. This is consistent with the experimental report that the WZ-BCT surface reconstruction was activated by the electron-beam irradiation.^48^ As the inner part of ZnO(101̅0) retains a conventional WZ structure based on six-atom rings, the outermost surface layer manifests a new lattice based on alternating four-atom and eight-atom rings, which is one characteristic of the BCT structure.

What is the origin of the surface phase transition from WZ to BCT? In order to answer this question, the density of states (DOS) of the WZ and BCT surfaces with 0.33 ML OVs were calculated, the result of which is shown in Figure 3d. The valence-band maximum (VBM) of BCT is slightly lower than that of WZ (0.20 eV), which is due to better stabilizing extra electrons produced by the removal of the oxygen. The geometric rearrangements caused by OVs are expected to be an exothermic process, as shown in the inset in Figure 3d. It is clear that the structural reorganization energy of the BCT surface is lower than that of the WZ surface and the difference is larger with the increasing OV concentration. In addition, we find that the degree of deformation caused by OV formation in the BCT surface, especially the shortening of the Zn–Zn bond, is larger than that of the WZ surface (by 0.03–0.09 Å), as shown in Table S3. Taken together, the ZnO(101̅0) surface tends to exist in the BCT surface in a low-oxygen chemical potential environment.

### 3.2 Geometric and Electronic Structures of the ZnO Surfaces

**Figure 4:**
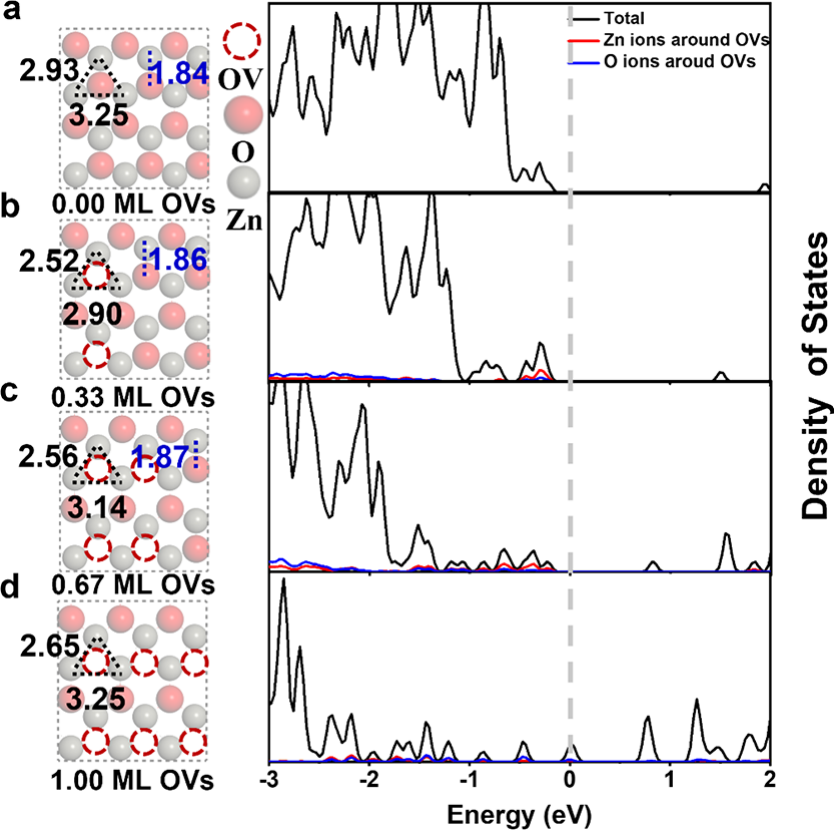
Geometric structure (left) (a) and local density of states (LDOS) (right) of ZnO(101̅0) with 0.33 ML (b), 0.67 ML (c), and 1.00 ML (d) OVs.

To understand the effects of the OVs, the geometric and electronic structures of the BCT surface with the OVs of 0.00, 0.33, 0.67, and 1.00 ML, including the local density of states (LDOS) and Bader charges, were calculated as a function of the OV concentration. Figure 4 illustrates the stable geometric structure and LDOS of the corresponding structures. Upon relaxation, the three neighboring Zn atoms of the surface O vacancy tend to form metal–metal bonds (2.93 Å → 2.52 Å) to reduce the total energy. Defective surfaces have shorter average Zn–Zn bonds, especially those with 0.33 ML OVs. In addition, the reduction in the concentration of surface oxygen atoms results in the Zn–O bond at the surface being increased slightly from 1.84 Å for the perfect surface to 1.87 Å for the surface with 0.67 ML OVs. The vacancy-induced distortions are observed to be mostly localized around the defect, whereas the geometry of the system is almost preserved in the regions far from the defect site, which is consistent with a finding in the literature.^49^

The LDOS in Figure 4 shows that the OVs have a significant effect on the electronic properties of ZnO(101̅0). The ZnO(101̅0) surface is a transparent semiconductor, and the OVs not only introduce defect states lying above the VBM but also narrow the band gap with an increasing concentration of OVs. Such defective states are mainly composed of the Zn 4s atomic orbitals near the vacancy, with a smaller contribution coming from the O 2p orbitals closest to the vacancy. The average Bader charges of ZnO(101̅0) in Table 1 demonstrates that the change in surface charge is much larger than the total charge change. Compared to the perfect surface, the defective surface with OVs has higher charges due to the electron transfer from the OVs to the Zn and remaining O atoms. The surface Bader charges of the Zn and O atoms increase with an increasing concentration of OVs, making it easier for Zn to lose electrons and more difficult for O to gain electrons.

**Table 1:** Average Bader Charges of ZnO(101̅0) with OVs^a^

	average Bader charge (e)			
(ML)	total Zn atoms	total O atoms	surface Zn atoms	surface O atoms
0.00	–1.17	1.17	–1.16	1.15
0.33	–1.12	1.17	–0.98	1.16
0.67	–1.07	1.16	–0.77	1.17
1.00	–1.02	1.16	–0.59	—

a Dash denotes that there is no such site on this catalyst.

### 3.3 Catalytic Activity of the ZnO Surfaces for H_2_ and CO Activation

As a widely used catalyst for methanol synthesis and the main component of the binary catalyst for light olefins from syngas, ZnO plays a great role in CO/H_2_ activation. In this work, we focus on the three main reaction steps, namely H_2_ cleavage, CO hydrogenation, and C–O bond cleavage, which are very important for product selectivity.^14^ The elementary reaction steps are as follows:

H_2_ reaction pathway:
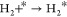
7
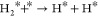
8

CO reaction pathway:
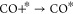
9
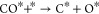
10
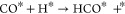
11
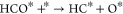
12where asterisks represents the ZnO(101̅0) surface.

#### 3.3.1 H_2_ and CO Adsorption

It is worth first discussing the adsorption structures and binding energies of H and CO on ZnO(101̅0) with various OV concentrations in Table 2. The OV site is defined as the newly generated active site that is a basic site due to the removal of oxygen atoms, and the other original sites including Zn_3c_, O_3c_, and Zn_3c_O_3c_ are defined as the intrinsic sites. We considered three modes of H adsorption, i.e., molecular H_2_, atomic H, and the 2H atoms as a result of H_2_ dissociative adsorption. As for the adsorption of H_2_, the chemisorption energies of H_2_ on the ZnO(101̅0) surface are only slightly negative (−0.05 eV). Compared to H on OV or Zn_3c_ sites to form hydride structures, the atomic H is more strongly adsorbed on an oxygen site as a proton. Note that the OV site can stabilize the hydrides better than the Zn_3c_ site with a relatively stronger adsorption energy. Surprisingly, the co-adsorption of 2H dramatically enhances the interaction of H atoms with the oxide surface due to a strong Lewis acid–base interaction.^50^ Namely, the adsorption of H (Lewis base) at the O site may enhance the ability of the oxide surface to donate electrons to the hydride (Lewis acid) at the Zn site, hence giving rise to stronger chemical bonding between the adsorbate and the surface.

**Table 2:** Adsorption Energies of H Species (H_2_/H/2H) and CO on the Intrinsic Site and the New OV Site^a^

	*E*_ads_ (eV)								
	H_2_		H			2H		CO	
(ML)	Zn_3c_	OV	O_3c_	Zn_3c_	OV	Zn_3c_O_3c_	OV	Zn_3c_	OV
0.00	–0.05	—	–0.09	1.86	—	–0.20	—	–0.27	—
0.33	–0.04	0.00	–0.01	1.67	0.27	–0.17	–0.03	–0.23	–0.16
0.67	–0.04	0.00	–0.12	1.34	–0.01	–0.16	–0.15	–0.21	–0.19
1.00	—	–0.01	—	—	–0.18	—	–0.09	—	–0.20

a Dashes denote that there is no such reaction site on this catalyst.

As for the adsorption of CO, we find that the intrinsic Zn_3c_ active sites acting as electron acceptors show a slightly stronger adsorption energy than the OV sites, which act as electron donors. Taken together, the newly generated OV sites are not conducive to the adsorptions of both H and CO.

**Figure 5:**
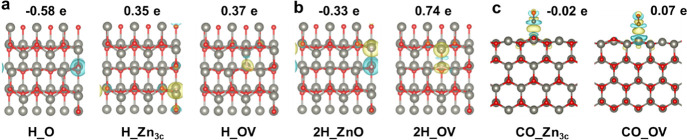
Likely adsorption configurations for H (a), 2H (b), and CO (c) on ZnO(101̅0) with 0.67 ML OVs and the corresponding charge density differences at an isosurface value of 5 × 10^–3^ eÅ^–3^. Yellow and blue bubbles represent charge accumulation and depletion, respectively.

The corresponding adsorption structures and the charge density differences induced by the adsorptions of various species on different sites are shown in Figure 5. Apparently, with the increasing amounts of the OVs, the OV sites as electron-donating bases increase the adsorption of all the species. The Zn_3c_ site acts as an electron-donating site for H but an electron-withdrawing site for CO species; therefore, different trends in adsorption energy can be seen as the concentration of the OVs increases. The O site and Zn_3c_O_3c_ site as an electron-withdrawing group for H species are unfavorable to the adsorption progress with the increasing concentration of OVs. These results can be understood as follows: Due to the extra electrons generated by the departure of the oxygen, the electron-donating ability of the surfaces to the adsorbates increase while the electron-withdrawing ability of the surface decrease with the increasing concentration of OVs. Collectively, H and CO are prone to adsorption on the intrinsic sites. The introduction of OVs alleviates the adsorption of H/CO on the intrinsic sites except H adsorption on the Zn_3c_ site while strengthening the adsorption of H/CO on the newly generated OV sites.

#### 3.3.2 H_2_ Dissociation, CO Hydrogenation, and C–O Dissociation

**Figure 6:**
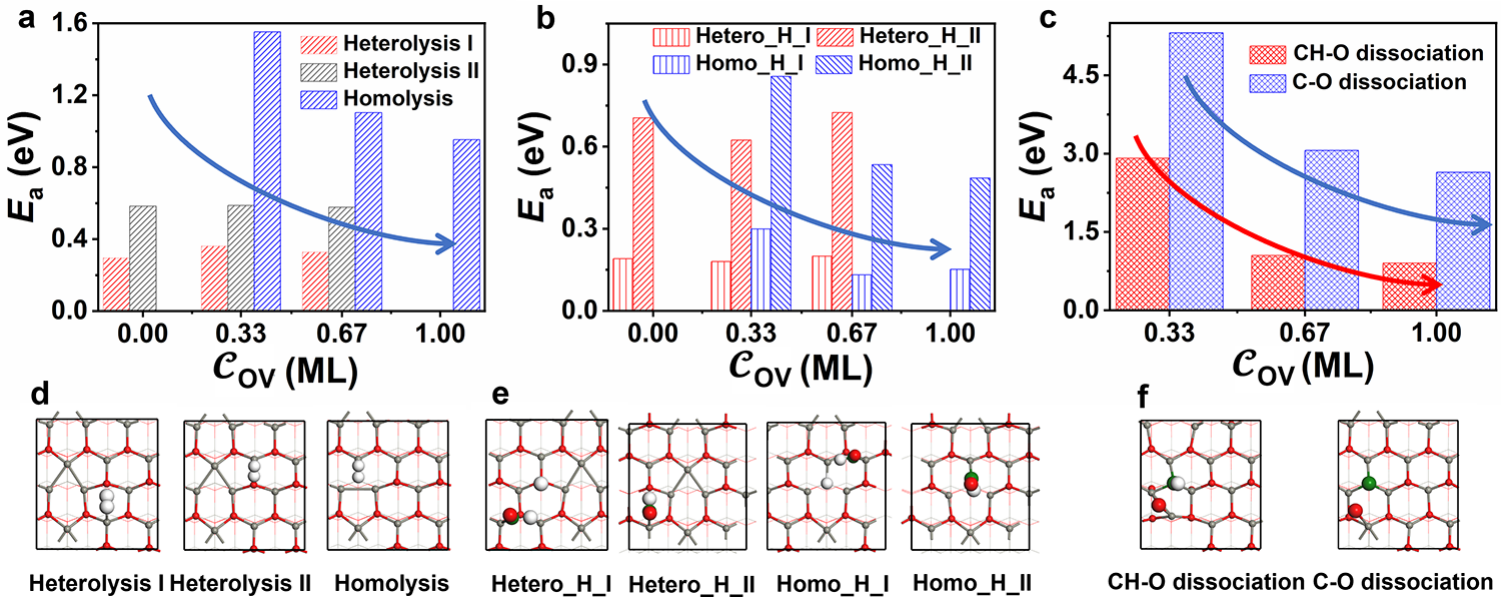
Comparison of the H_2_ dissociation barrier (a), CO hydrogenation barrier (b), and C–O dissociation barrier (c) on ZnO(101̅0) with various concentrations of OVs. The corresponding configurations of H_2_ dissociation (d), CO hydrogenation (e), and C–O dissociation (f) on ZnO(101̅0) with 0.33 ML OVs for illustration purposes. Some reaction pathways do not occur at some OV concentrations and therefore are not shown in the figure.

As H_2_ cleavage, CO hydrogenation, and C–O dissociation are the three important elementary steps in the synthesis gas conversion on oxide surface,^14,19^ we discuss the effects of OV on these three steps below (Figure 6).

##### 3.3.2.1 H_2_ → H + H

There are two main reaction pathways for H_2_ dissociation on the stoichiometric surface (Figure 6a,d). Heterolysis I represents the H_2_ dissociation on two neighboring Zn–O pairs, while heterolysis II denotes the H_2_ dissociation on one single Zn–O pair. Clearly, heterolysis I is kinetically preferred, being consistent with the literature reports^51,52^ (0.29 eV vs 0.58 eV). When introducing OVs, H_2_ homolytic dissociation will take place on the OV site with a relatively high barrier (1.55 eV on the ZnO surface with 0.33 ML OVs). We also studied the homolysis of H_2_ on the O site of the intact surface and found that the energy barrier was as high as 2.19 eV. As a function of OV concentration, heterolytic dissociation reactivity at the intrinsic active sites is almost unchanged, while homolytic dissociation reactivity at the OVs increases gradually.

##### 3.3.2.2 CO + H → HCO

We investigated the reactivity trend of CO hydrogenation by calculating the reaction of the adsorbed CO with the various kinds of surface H species, generating HCO on the ZnO(101̅0) surface. The CO hydrogenation with the product of homolysis and heterolysis I due to the low dissociation barrier compared to the heterolysis II were considered in this section. The CO hydrogenation reaction by H on the Zn and oxygen sites obtained by heterolysis I are defined as hetero_H_I and hetero_H_II, while that on the Zn and OV sites obtained by homolysis are denoted as homo_H_I and homo_H_II. Some intriguing results are found, which are shown in Figure 6b,e: First, the CO hydrogenation reactivity by H on the Zn site (hetero_H_I and homo_H_I) is two to three times higher than that of the CO hydrogenation reactivity by H on the O site (hetero_H_II) and OV site (homo_H_II) due to the weak adsorption of H on the Zn sites. Second, with the increase in the OV concentration, the CO hydrogenation reactivity by H from hetero-cleavage at the intrinsic active sites is hardly affected, while that from homo-cleavage at the newly generated OV active site increases.

##### 3.3.2.3 CO → C + O and CHO → CH + O

We studied both the direct C–O bond breaking into C and O as well as the H-assisted C–O bond cleavage (Figure 6c,f). On the perfect ZnO(101̅0) surface, the C–O bond cleavage is impossible, while the presence of OVs facilitates greatly the reaction. It is found that the barrier of the H-assisted C–O bond cleavage is significantly lower than that of the direct C–O bonding breaking. With the increasing OV concentration, the energy barrier of C–O activation decreases, especially by 2.25 eV from the surface with 0.33 ML OVs to the surface with 0.67 ML OVs.

Overall, the effect of OVs on H_2_ and CO activation is mainly reflected in the following two aspects: One is of geometrical nature, and the other is of electronic properties. First, the geometrical effect of OVs is the creation of Zn cluster-like reaction sites that do not exist on the stoichiometric surface. A dramatic barrier decrease for C–O dissociation and a relatively high barrier for H_2_ homolysis and CO hydrogenation can be seen. Second, the electronic effect is due to the presence of excess electrons on the surface, which affects the adsorption of reactants and reaction intermediates, thereby influencing the catalytic reaction. Since the electrons and deformations generated by the OVs are localized near the OVs, as the OV concentration increases, the activity of the intrinsic sites is hardly affected, while that of the newly generated OV site increases gradually. A similar activity trend with the increase of the OV concentration can be observed on the WZ surface, and more details are provided in the SI (Tables S4−S8).

#### 3.3.3 Effects of Oxygen Vacancy Distribution on the Activities of the ZnO Surfaces

**Figure 7:**
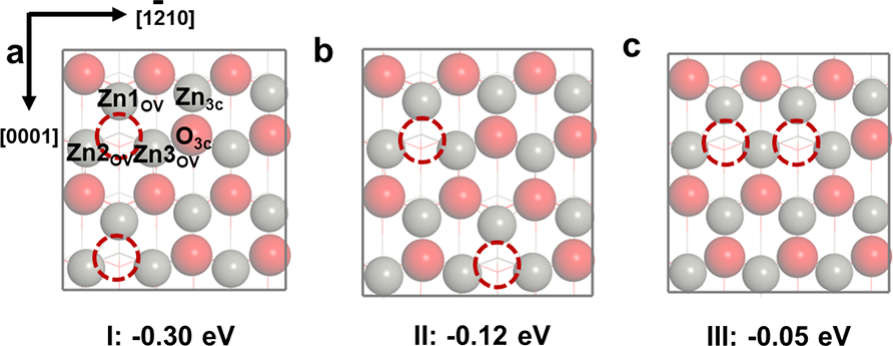
The distribution of OVs on the surface with 0.33 ML OVs tending together along the [0001] direction(a), forming at isolated locations away from each other (b) and along the [12̅10] direction (c).

In this section, we address the following question: How does the distribution of the OVs influence the reaction activity? As mentioned above, the ZnO(101̅0) surface with 0.33 ML OVs is the most likely structure under the experimental conditions. Therefore, we select the ZnO(101̅0) surface with 0.33 ML OVs as a simple model to investigate the distribution of the OVs. In such a model, there are mainly three ways of arranging OVs: (i) clustering along the [0001] direction; (ii) locating at isolated locations; and (iii) clustering along the [12̅10] direction (Figure 7). Our calculations show clearly that the OVs tend to gather along the [0001] direction with a lower energy.

We investigated H_2_ dissociation, CO hydrogenation, and CH–O dissociation on the ZnO(101̅0) surfaces with these OVs distributions. Some interesting results were obtained as shown in Table 3 and Figure S6. First, the distribution of the OVs has little effect on the reactions that take place on the intrinsic active sites including H_2_ heterolysis I and CO hydrogenation. However, for reactions on the OV sites, the OVs clustering along [12̅10] can remarkably reduce the reaction barriers, especially for the C–O dissociation. As shown in Figure S6a,b, OVs connecting together can stabilize the cleavage product very well, reducing the reaction barriers of H_2_ homolytic dissociation (1.55 eV → 1.19 eV) and CH–O dissociation (2.91 eV → 1.22 eV). Secondly, the newly generated Zn1_OV_ site (see Figure 7) has an interesting influence on H_2_ heterolytic dissociation and CO hydrogenation. The Zn1_OV_ site with fewer positive charges in comparison to the Zn_3c_ site weakens the polarization of the H_2_ heterolytic dissociation, thereby increasing the cleavage barrier by 0.40 eV (Figure S6c). Meanwhile, a relative low CO hydrogenation barrier can be achieved when the CO on the Zn_3c_ site is attacked by the atomic H on the Zn1_OV_ site (Figure S6d).

**Table 3:** Energy Barriers of Various Reactions on ZnO(101̅0) with Various OVs Distributions at 0.33 ML OVs

		*E*_a_ (eV)		
reaction types	active sites	configuration I	configuration II	configuration III
heterolysis I	Zn_3c__O_3c_	0.36	0.33	0.28
	Zn1_OV__O_3c_	—	0.66	0.68
homolysis	OV	1.55	1.42	1.19
hetero_H_I^a^	Zn1_OV_ _Zn_3c_	0.20	0.21	0.19
	Zn_3c__Zn_3c_	0.18	0.16	0.17
	Zn_3c__ Zn1_OV_	—	0.01	0.05
	Zn1_OV__Zn1_OV_	—	—	0.18
hetero_H_II	Zn_3c__O_3c_	0.62	0.59	0.70
	Zn1_OV_ _O_3c_	—	0.72	0.81
CH–O dissociation	OV	2.91	3.98	1.22

a For CO hydrogenation, the active site A_B means the CO adsorption on the A site and the H adsorption on the B site.

Overall, the influence of the arrangement of OVs on the ZnO(101̅0) surfaces for H_2_/CO activation is mainly manifested in the following two aspects; (i) significantly decreasing the energy barriers of H_2_ homolysis and C–O bond dissociation on the OV sites, especially for the C–O bond activation when OVs connect together along the [12̅10] direction, and (ii) creating a new reaction site Zn1_OV_, increasing the reactivity for CO hydrogenation while decreasing the reactivity for H_2_ heterolysis.

#### 3.3.4 Difference between the Activity of the Two Surface Phases

**Figure 8:**
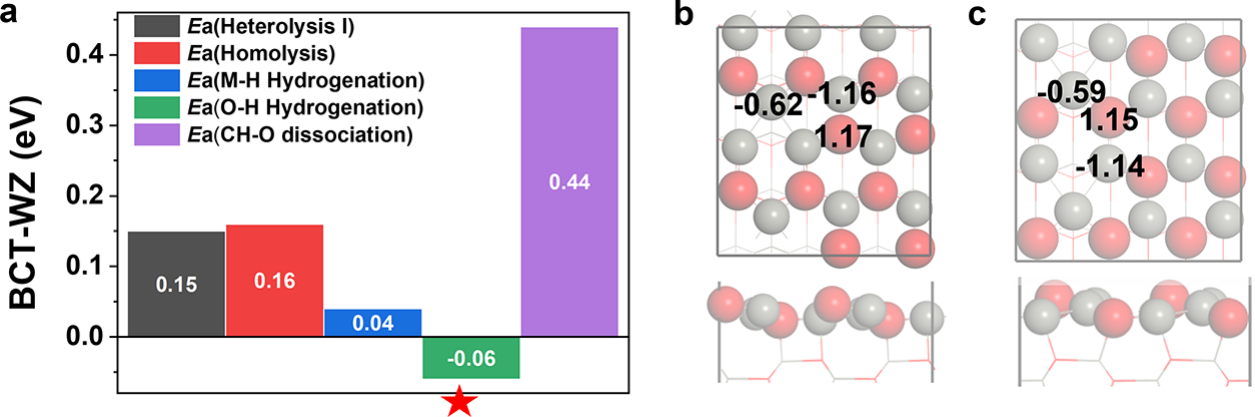
Energy barrier differences for various reactions of the two surface with 0.33 ML OVs (a). Bader charge analysis of the BCT surface (b) and WZ surface (c) with 0.33 ML OVs.

In this section, we present the results of the investigation on what effects the original WZ surface and the BCT surface have on the activation of H_2_ and CO, in which the BCT surface is a more stable surface as a result of the phase transformation during the generation of OVs. From Figure 8a, one can see that the WZ surface exhibits slightly better catalytic activity except for the CO hydrogenation reaction, in which H is on the oxygen site. In other words, the metastable WZ surface exhibits relatively good activity. In comparison to the WZ surface, the Zn_3c_–O_3c_ bond of the BCT surface is more stably derived from the slightly shorter Zn_3c_–O_3c_ bond (0.005 Å) and more Bader charges (0.01 e), thus weakening the adsorption of intermediates on the Zn_3c_O_3c_ site (Figure 8b,c) and lowering the reaction barrier. The activities of the WZ surface with OVs of 0.00, 0.67, and 1.00 ML were also investigated, which are reported in the SI. By comparing the results from BTC and WZ surfaces, we find that the WZ surface has a lower adsorption energy of reactants and intermediates and the activity is relatively high except for the CO hydrogenation by H sitting on the O site.

## 4 Conclusions

In summary, we investigated the effect of oxygen vacancies on the catalytic activity of ZnO(101̅0) for CO/H_2_ activation by a combination of machine learning techniques, genetic algorithm-based structural searches, and DFT calculations. The following results are obtained:

(1) Surface structural transition from the WZ to BCT phases can be seen in the presence of OVs. The surface with 0.33 ML OVs is the most stable surface under experimental conditions.
(2) The excess electrons brought by the departure of oxygen are enriched around OV, reducing the band gap of ZnO(101̅0). With the increasing concentrations of OVs, the adsorption of CO and H species on the intrinsic active sites gradually decrease except for the adsorption of H on Zn, while the adsorptions on the OV reaction sites are enhanced.
(3) The most important impact of OV is the generation of an OV site composed of three Zn ions, which enables H_2_ homolysis and C–O bond dissociation. In addition, intrinsic sites exhibit nearly unchanged activity, while OV sites exhibit elevated activity with an increasing number of OVs. Moreover, the arrangement of OVs significantly affects the reactivity of the C–O bond cleavage.
(4) The metastable WZ surface exhibits relatively good activity except for the CO hydrogenation reaction of the H on the oxygen site.

These results strengthen the fundamental understanding of the oxygen vacancy for the CO and H_2_ activation process on ZnO surfaces, and these understandings may offer a strategy to rationally design metal oxide catalysts for CO/H_2_ activation reactions through creating local metal clusters by removing oxygens and controlling its concentration.

## Supporting Information Available

Details of the GA global optimization accelerated by the MLPs and oxygen chemical potential calculations; full list of datasets; Δ*G*_f_ for unit cells of different sizes and symmetries; optimized structures and energy barriers on the WZ and BCT surfaces (PDF)
