# Supplementary material for: Comprehensive Study of Oxygen Vacancies on the Catalytic Performance of ZnO for CO/H2 Activation Using Machine Learning-Accelerated First-Principles Simulations
Source: ACS Catal. 2023 Mar 30;13(8):5104–13. doi: 10.1021/acscatal.3c00658 (PMC10127212; doi:10.1021/acscatal.3c00658)
Supplement: Supplementary file 1 — cs3c00658_si_001.pdf [file cs3c00658_si_001.pdf]

Supporting Information

Comprehensive Study of Oxygen Vacancies on the Catalytic Performance of ZnO for CO/H_2 Activation Using Machine Learning-accelerated First-principles Simulations

Yulan Han, Jiayan Xu, Wenbo Xie, Zhuozheng Wang, P. Hu*

School of Chemistry and Chemical Engineering, Queen's University Belfast, Belfast BT9 5AG, UK

Corresponding Author

*E-mail address: p.hu@qub.ac.uk

Table of Contents

1. Computational Methods 2

1.1 Active Learning for Accelerating Genetic Algorithms (GAs) Global Optimization 2

1.2 Oxygen Chemical Potential (μ_O) Calculation 6

2. Stability of the ZnO Surfaces 7

3. Geometric and Electronic Structures of the ZnO Surfaces 9

4. Catalytic activities of the Body-centered-tetragonal (BCT) and Wurtzite (WZ) Surfaces with Various Concentrations of OVs 10

4.1 H_2/CO Adsorption 12

4.2 H_2 Dissociation 12

4.3 CO Hydrogenation 12

4.4 C-O Activation 13

5. References 50

1. Computational Methods

1.1 Active Learning for Accelerating Genetic Algorithms (GAs) Global Optimization

Supplemental Note S1. GAs are metaheuristic optimization algorithms inspired by Darwinian evolution. Performing crossover, mutation, and selection operations, the algorithm progresses a population of evolving candidate solutions.

To estimate whether a configuration should be selected and added to the training dataset, two selection procedures are employed. First, the energy uncertainty assessed by the model committee falls between the bounds ($\sigma_{lo.} < \sigma_t < \sigma_{hi.}$), which indicates that the corresponding structure is not well learned and rules out the unphysical configurations. In this strategy, an ensemble of models is concurrently trained with different random seeds that initialize the network parameter and the prediction uncertainty is estimated by the variance of these model outputs. For instance, the mean and variance of the total energy prediction are given by

$$\bar{E} = \frac{1}{M}\sum_1^M E^i \quad (1)$$

$$\sigma^2(E) = \frac{1}{M-1}\sum_1^M \left|E^i - \bar{E}\right|^2 \quad (2)$$

where M is the number of models. This estimation can be used for other properties as well, such as forces and stresses. Due to the high nonlinearity of the neural network model and the randomness of the optimizer, the final converged models would be slightly different even with the same inputs. For $\sigma_t < \sigma_{lo.}$, this means that the explored configurations can be predicted with a high accuracy by the Embedded atom neural network(EANN) models. Labelling these configurations does not help to improve the quality of the model but leads to a waste of the computational resources. $\sigma_t > \sigma_{hi}$ illustrates that the simulation sometimes samples unphysical configurations, e.g., overlapping atoms. These configurations may suffer from difficulties in converging self-consistent iterations and are irrelevant to the accuracy of the final model. Therefore, structures corresponding to this case ($\sigma_{lo.} < \sigma_t < \sigma_{hi.}$) are selected as candidates for labelling.

Second, the CUR decomposition method was used to further sift the candidates based on their structural diversity.[1-2] Here, we use a configuration-averaged Smooth Overlap of Atomic Positions (SOAP) descriptor that compares entire unit cells to one another to quantify the difference between candidate structures. Screening of high-quality structures will greatly improve the quality of potential energy surfaces (PESs), thereby accelerating structure searching effectively.

The workflow has two stop criteria. First, the energy deviation of the explored minima is less than 0.02eV/atom to ensure the accuracy of the PES. Second, the same lowest-energy structure is produced during several consecutive GA optimization processes. Although the EANN model can generate energies and forces that are very close to the density functional theory (DFT) reference model, a small intrinsic error still exists. Therefore, the structures with sufficiently low energies (the top 100 most stable structures for the same composition) were relaxed with DFT. These structures converged very fast within 10-20 ionic steps, indicating well-trained machine learning potentials (MLPs).

Table S1. Energy and force root mean squared error (RMSE) on DFT-labelled structures. The Nframes is the number of the training structures, as distinguished by the chemical formula (system) and the number of atoms per cell (Natoms).

System	Natoms	Nframes	E_{RMSE} (eV/atom)	F^{o}_{RMSE} (eV/Å)	F^{Zn}_{RMSE} (eV/Å)
O0Zn14	14	25	0.0263	nan	0.0594
O0Zn15	15	683	0.0212	nan	0.0945
O0Zn16	16	4164	0.0179	nan	0.1045
O0Zn17	17	8	0.0121	nan	0.0428
O0Zn19	19	63	0.023	nan	0.0895
O0Zn24	24	229	0.0185	nan	0.0548
O0Zn28	28	22	0.019	nan	0.0529
O0Zn29	29	18	0.0203	nan	0.1185
O0Zn30	30	56	0.0257	nan	0.1138
O0Zn31	31	67	0.0129	nan	0.0612
O0Zn32	32	87	0.017	nan	0.0529

O10Zn10	20	1411	0.0102	0.102	0.0963
O10Zn16	26	2604	0.0106	0.1175	0.13
O10Zn24	34	334	0.0147	0.1578	0.1323
O11Zn0	11	790	0.039	0.3809	Nan
O11Zn15	26	28	0.0149	0.1697	0.1745
O11Zn16	27	3017	0.0195	0.1426	0.1435
O12Zn16	28	3339	0.026	0.1588	0.1545
O13Zn15	28	231	0.0158	0.1383	0.1441
O13Zn16	29	3143	0.0811	0.531	0.5044
O14Zn15	29	53	0.0166	0.1467	0.1414
O14Zn16	30	2765	0.1102	0.2619	0.2041
O15Zn16	31	3234	0.015	0.1929	0.1596
O16Zn14	30	341	0.0933	0.2363	0.1821
O16Zn16	32	2337	0.0223	0.3803	0.3455
O2Zn17	19	250	0.017	0.1463	0.1002
O4Zn0	4	79	0.0509	0.5566	Nan
O6Zn4	10	40	0.0811	0.3265	0.2949
O6Zn6	12	1415	0.022	0.205	0.194
O6Zn8	14	306	0.0266	0.1843	0.1749
O7Zn16	23	33	0.0212	0.15	0.1378
O7Zn8	15	309	0.0284	0.2006	0.1864
O8Zn16	24	1129	0.0067	0.0606	0.0867
O8Zn8	16	3588	0.0149	0.1055	0.0862
O9Zn16	25	1445	0.0084	0.0871	0.1041
O36Zn48	84	1428	0.0043	0.0772	0.0918
O37Zn48	85	1254	0.029	0.099	0.1049
O38Zn48	86	1433	0.0048	0.0915	0.1082
O39Zn48	87	1452	0.0122	0.1012	0.1136
O40Zn48	88	1428	0.0092	0.1156	0.1186
O41Zn48	89	1357	0.0125	0.1386	0.1314
O42Zn48	90	1414	0.0063	0.1295	0.126
O43Zn48	91	1440	0.0144	0.1321	0.1287
O44Zn48	92	1451	0.0225	0.1981	0.1351
O45Zn48	93	1369	0.0178	0.1434	0.1369
O46Zn48	94	1339	0.0191	0.1622	0.1319

O47Zn48	95	1397	0.0215	0.167	0.1371
O48Zn48	96	1359	0.0193	0.176	0.1431
ALL	nan	55764	0.0363	0.1917	0.1673

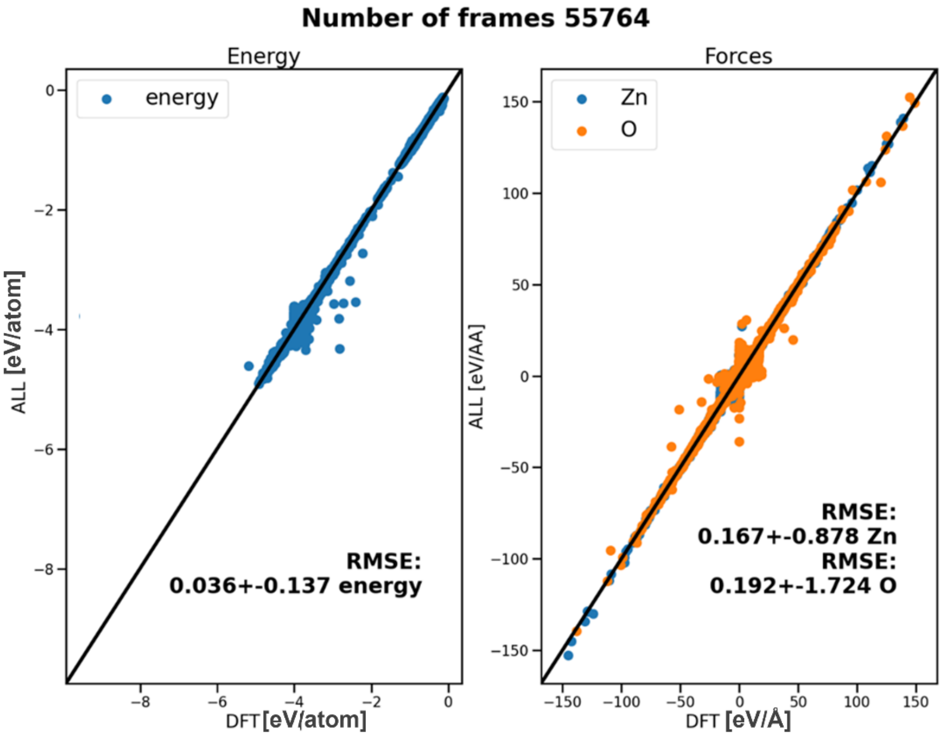

Figure S1. Energy and force RMSE on DFT-labelled structures. The horizontal and vertical axes of Figure S1a are the energies per atom (eV/atom) calculated from DFT and EANNP, respectively. The fitting error (RMSE) of the energy predicted by the EANNP is 0.036 eV/atom. The RMSE in forces is 0.192 eV/Å for O and 0.167 eV/Å for Zn. In the process of preparing the training data, some unstable structures with bond distances being relatively largely different from the optimized ones for training the potential.

1.2 Oxygen Chemical Potential (μ_O) Calculation

Table S2. Calculated energy, the thermal correction to Gibbs Free energy and Gibbs free energy (eV).

	E_{DFT}	ZPE	$U(T)$	$TS(T)$	$k_BTln(\frac{P}{P_0})$	$\mu(T,P)$
H_2O(673 K,0.01 Kpa)	-14.22	0.58	0.25	1.53	-0.53	-15.45
H_2(673 K,1.5 Mpa)	-6.76	0.27	0.21	1.09	0.16	-7.20
CO_2(673K,0.0765 Mpa)	-22.99	0.32	0.28	1.75	-0.02	-24.16
CO(673 K,1 Mpa)	-14.80	0.14	0.21	1.56	0.13	-15.88

Supplemental Note S2. For H_2-assisted OV generation, the pressure of H_2O is set to 0.01 Kpa according to the experiment.[3-4] For CO-assisted OV generation, the equilibrium pressure of CO_2 is evaluated at 673 K, 0.0765 Mpa, according to the experimental results (CO conversation at ~17% and CO_2 selectivity at ~45%). [5] Based on the above calculations for μ_O under the experimental conditions(-8.25 eV and -8.28 eV when the surface is equilibrium with H_2 and CO, respectively). The μ_O under the standard condition(298.15K, 0.1 Mpa) is -5.15eV. Therefore, the $\Delta\mu_O =$ -3.10 eV and -3.13 eV when the surface is equilibrium with H_2 and CO, respectively.

2. Stability of the ZnO Surfaces

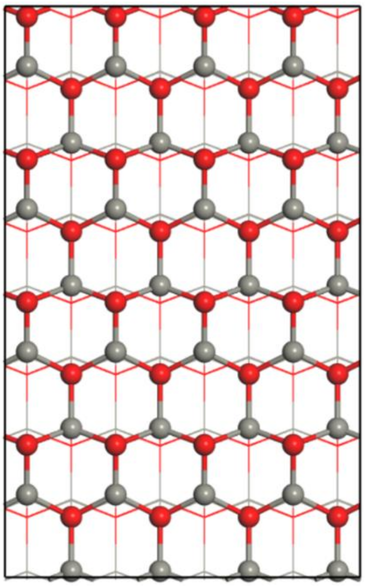

Figure S2. Top view of p(4x4) ZnO surface.

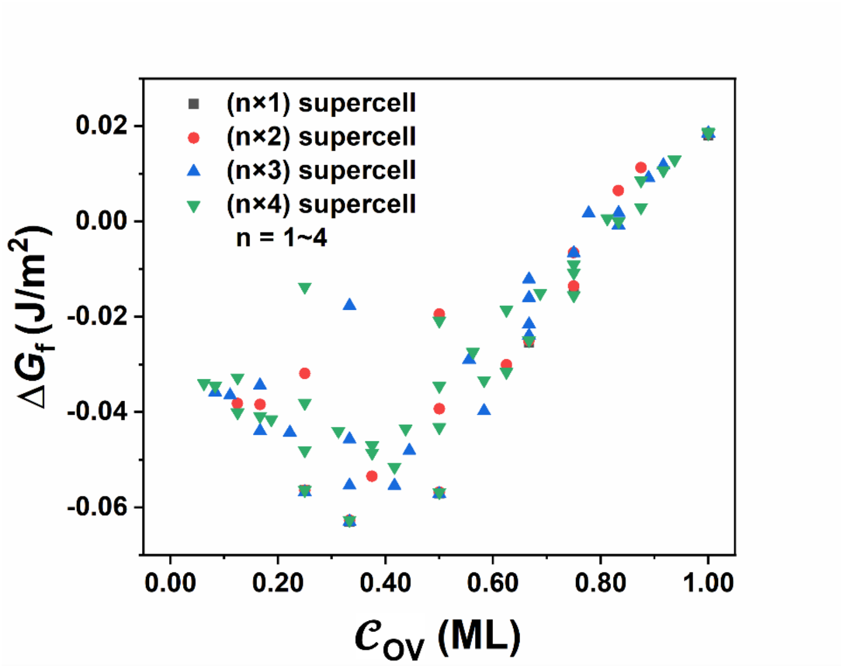

Figure S3. OV formation energies of ZnO $(10\bar{1}0)$ at different $\mathcal{C}_{OV}$ referring to the perfect ZnO$(10\bar{1}0)$ surface under the CO reduction atmosphere (673K, 1Mpa).

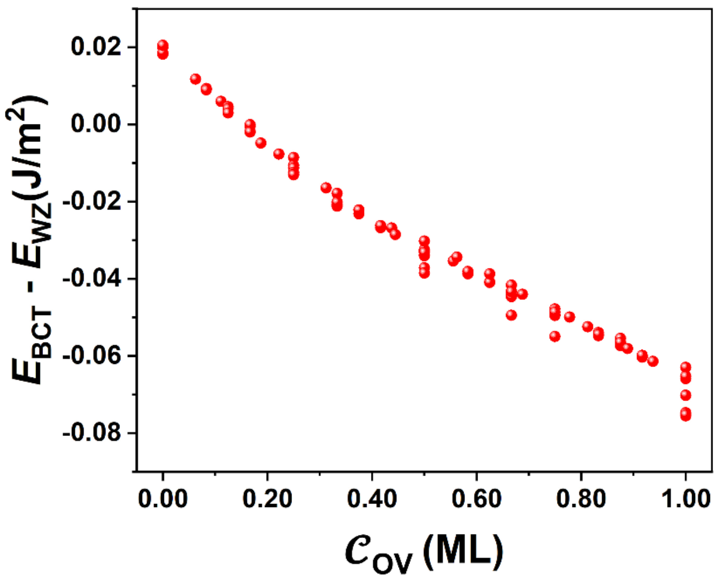

Figure S4. The energy difference between the BCT surface and WZ surface as a function of OV concentrations.

Supplemental Note S3. To take the unit cells of different sizes and symmetries into consideration, we have explored the surfaces with a number of OVs using unit cells of $p(1\times1)$, $p(2\times1)$, $p(3\times1)$, $p(4\times1)$, $p(1\times2)$, $p(2\times2)$, $p(3\times2)$, $p(4\times2)$, $p(1\times3)$, $p(2\times3)$, $p(3\times3)$, $p(4\times3)$, $p(1\times4)$, $p(2\times4)$, $p(3\times4)$, $p(4\times4)$. Firstly, the largest unit cell, $p(4\times4)$ ZnO surface with 256 numbers of atoms, is large enough, containing 4 O atoms in each row and column on the top layer, respectively, as shown in **Figure S2**. Secondly, they cover a range of surfaces with different symmetries. These warrant that the data contain general features of the ZnO surface. To ensure the energy convergency of models of various sizes, high density K-points were used. The KSPACING was set to 0.167 $Å^{-1}$. In order to compare the OV formation energies from different unit sizes, they are converted to the unit of J m^{-2}. As shown in **Figure S3**, the surface with 0.33 ML OVs is the most likely surface under the experimental conditions. And with the increasing concentration of OVs, the energy difference between the BCT surface and WZ surface increase in **Figure S4**.

3. Geometric and Electronic Structures of the ZnO Surfaces

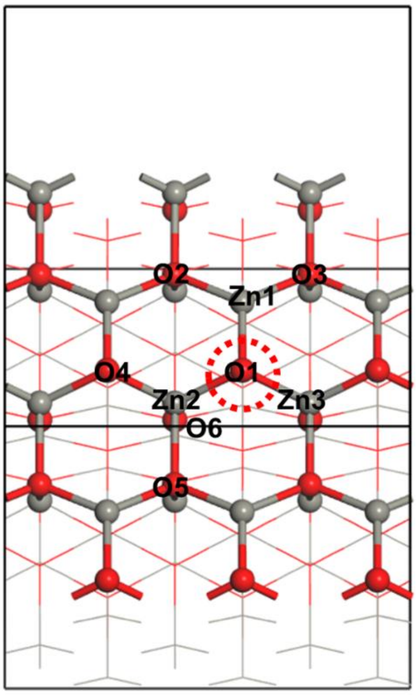

Figure S5. Structure of the optimized BCT surface. Key bond lengths and Zn-Zn pair distances before and after introducing an OV defect at the O1 site are listed in **Table S3**.

Table S3. Computed key atomic distances (Å) for the defect-free BCT and WZ before and after introducing an OV. The labels of atoms are shown in **Figure S5**.

	BCT	BCT with 0.17 ML OVs	△BCT	WZ	WZ with 0.17 ML OVs	△WZ
Zn1-Zn2	2.93	2.50	-0.43	2.97	2.57	-0.40
Zn1-Zn3	2.94	2.51	-0.43	2.97	2.57	-0.40
Zn2-Zn3	3.25	2.95	-0.31	3.25	3.03	-0.22
Zn2-O1	1.90	nan	nan	1.92	nan	nan
Zn2-O4	1.90	1.94	0.03	1.92	1.94	0.02
Zn2-O5	2.00	2.00	0.00	1.99	1.99	0.00
Zn2-O6	2.08	2.07	-0.01	2.07	2.06	-0.01
Zn1-O1	1.84	nan	nan	1.85	nan	Nan
Zn1-O2	1.90	1.95	0.05	1.92	1.99	0.07
Zn1-O3	1.90	1.95	0.05	1.92	1.98	0.06

4. Catalytic Activities of the Body-centered-tetragonal (BCT) and Wurtzite (WZ) Surfaces with Various Concentrations of OVs

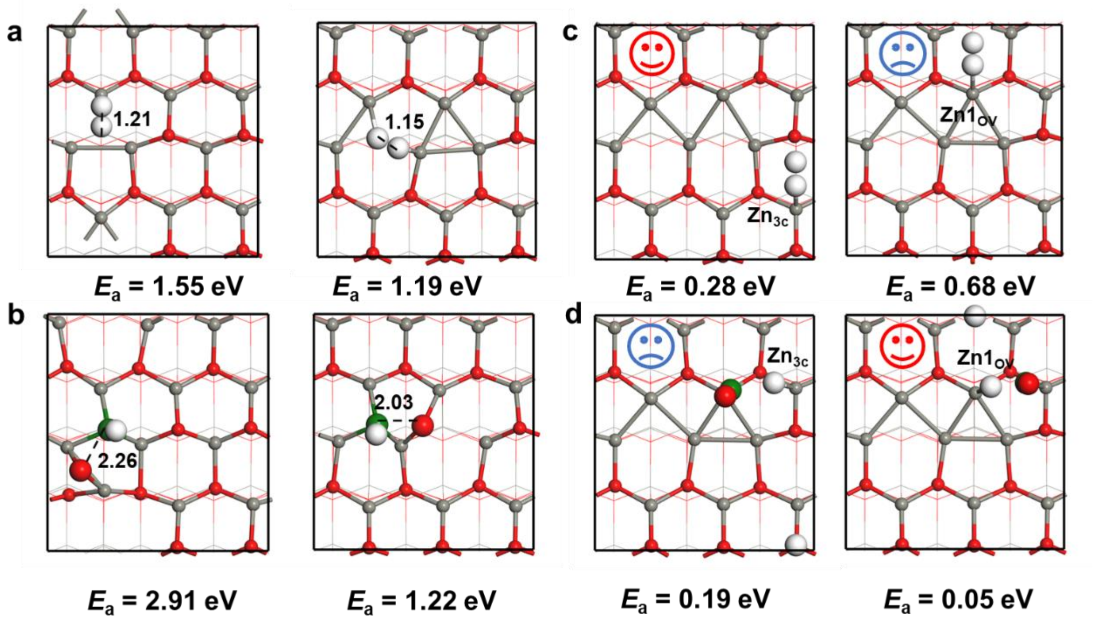

Figure S6. The configurations of the H_2 homolytic dissociation (a) and CH-O dissociation(b) on model I and model III. (c) The configurations of the heterolysis I on the Zn_{3c}_O_{3c} and $Zn1_{OV}$_O_{3c} sites. (d) The configurations of the CO hydrogenation hetero_H_I on $Zn1_{OV}$_Zn_{3c} and Zn_{3c}_$Zn1_{OV}$ sites.

Table S4. Calculated the adsorption energy of H_2 and CO on BCT and WZ surface with various concentrations of OVs.

	BCT surface		WZ surface	
	CO (eV)	H_2 (eV)	CO (eV)	H_2 (eV)
0.00 ML	-0.27	-0.05	-0.38	-0.13
0.33 ML	-0.23	-0.04	-0.31	-0.08
0.67 ML	-0.21	-0.04	-0.27	-0.06
1.00 ML	-0.20	-0.01	-0.33	-0.06

Table S5. Calculated energy barriers and enthalpy changes of H_2 dissociation on BCT surface with various concentrations of OVs.

	Heterolysis I		Heterolysis II		Homolysis	
	E_a (eV)	$\triangle H$ (eV)	E_a (eV)	$\triangle H$ (eV)	E_a (eV)	$\triangle H$ (eV)
0.00 ML	0.29	0.06	0.58	-0.20		
0.33 ML	0.36	0.07	0.59	-0.17	1.55	-0.03
0.67 ML	0.33	0.05	0.58	-0.16	1.10	-0.15
1.00 ML					0.95	-0.09

Table S6. Calculated energy barriers and enthalpy changes of H_2 dissociation on WZ surface with various concentrations of OVs.

	Heterolysis I		Heterolysis II		Homolysis	
	E_a (eV)	$\triangle H$ (eV)	E_a (eV)	$\triangle H$ (eV)	E_a (eV)	$\triangle H$ (eV)
0.00 ML	0.19	-0.21	0.54	-0.45		
0.33 ML	0.21	-0.15	0.53	-0.38	1.39	-0.33
0.67 ML	0.24	-0.11	0.51	-0.32	1.07	-0.39
1.00 ML					0.92	-0.35

Table S7. Calculated energy barriers of CO hydrogenation on BCT and WZ surface with various concentrations of OVs.

	BCT surface				WZ surface			
	Hetero _H_I (eV)	Hetero _H_II (eV)	Homo _H_I (eV)	Homo _H_II (eV)	Hetero _H_I (eV)	Hetero _H_II (eV)	Homo _H_I (eV)	Homo _H_II (eV)
0.00 ML	0.19	0.71			0.19	0.79		
0.33 ML	0.18	0.62	0.30	0.86	0.14	0.68	0.28	0.70
0.67 ML	0.20	0.71	0.13	0.53	0.16	0.72	0.12	0.56
1.00 ML			0.15	0.49			0.17	0.39

Table S8. Calculated energy barriers of CO breakage on BCT and WZ surface with various concentrations of OVs.

	BCT surface		WZ surface	
	C-O dissociation (eV)	CH-O dissociation (eV)	C-O dissociation (eV)	CH-O dissociation (eV)
0.33 ML	5.31	2.91	4.42	2.47
0.67 ML	3.06	1.05	2.71	0.81
1.00 ML	2.64	0.90	2.44	0.71

Supplemental Note S4. We have also investigated the catalytic activities of the WZ surface with various concentrations of OVs, including H_2/CO adsorption, H_2 dissociation, CO hydrogenation and C-O dissociation.

4.1 H_2/CO Adsorption

As shown in **Table S4**, CO shows stronger chemical adsorption than H_2. With the increasing of the OVs, the adsorption of CO on the WZ surface decreases, which is in line with the report of the BCT surface in the main text. It is worth noting that the WZ surface has stronger adsorption capacity for CO and H_2 than BCT surface because of the higher d band center of the WZ surface (-5.77 eV *vs.* -6.03 eV).

4.2 H_2 Dissociation

As shown in **Table S6**, with the increase of OV concentration, the reactivity of hetero-cleavage of H_2 at the intrinsic site hardly changed, but the reactivity of homo-cleavage of H_2 at the newly generated OV site increased gradually. Similarly, the WZ surface show better H_2 dissociation activity than the BCT surface.

4.3 CO Hydrogenation

As shown in **Table S7**, with the increase of OV concentration, the hydrogenation reactivity with H from heterolysis at the intrinsic reactive sites(O_{3c} and Zn_{3c}) is hardly affected while that from homolysis at the newly generated OV reactive site increases. Compared to the BCT surface, better CO hydrogenation reactivity can be seen except for the H sitting on the oxygen site.

4.4 C-O Activation

As shown in **Table S8**, the C-O bond is more easily broken as the concentration of OVs increases. A better C-O bond activation activity can be seen on the WZ surface.

Overall, as the concentration of OVs increases, a similar behavior trend can be seen on the BCT and WZ surface. That is, the activity occurring on the intrinsic site changes little while the activity occurring on the OV gradually increases. The metastable WZ surface shows better catalytic activity compared to the BCT surface except for the CO hydrogenation reactivity with the hydrogen adsorbed on the oxygen.

Optimized XYZ position for the transition states of H_2 heterolysis I on ZnO BCT surface with 0.33 ML OVs

AutoCreatByScript
1.00000000000000
9.7516200000000008 0.0000000000000000 0.0000000000000000
0.0000000000000000 10.4606600000000007 0.0000000000000000
0.0000000000000000 0.0000000000000000 24.3835499999999996
H O Zn
2 46 48
Direct
0.5794563691056167 0.4072243412552114 0.4283745722326627
0.5803411322933714 0.3257721769429525 0.4483508748627591
0.2534539999999978 0.2088630000000009 0.0123030000000028
0.5867870000000012 0.2088630000000009 0.0123030000000028
0.9201199999999973 0.2088630000000009 0.0123030000000028
0.2534539999999978 0.7088630000000009 0.0123030000000028
0.5867870000000012 0.7088630000000009 0.0123030000000028
0.9201199999999973 0.7088630000000009 0.0123030000000028
1.0867859999999965 0.4588630000000009 0.0507869999999997
0.4201199999999972 0.4588630000000009 0.0507869999999997
0.7534530000000004 0.4588630000000009 0.0507869999999997
1.0867859999999965 -0.0411369999999992 0.0507869999999997
0.4201199999999972 -0.0411369999999992 0.0507869999999997
0.7534530000000004 -0.0411369999999992 0.0507869999999997
1.0858016819270346 0.2116125333686435 0.1289910533621758
0.4208134764279651 0.2115195462046242 0.1287308552986575
0.7534547804362631 0.2115039897017647 0.1289450269139011
1.0857266100692891 0.7116739086357894 0.1291582807417617

0.4210128864851237	0.7116588378383538	0.1289482468694068
0.7532811174434554	0.7116234028995488	0.1291915977560263
0.5871111436142508	0.4619982790967059	0.1652254083064379
0.2537269999967597	0.4613986733292224	0.1637990076148176
0.9198248807479761	0.4618134794603986	0.1654351576404245
0.9196039554254717	-0.0383203333811766	0.1656691257483322
0.2537416194854472	-0.0384586980094468	0.1639486208969449
0.5870051428586915	-0.0384038634645466	0.1655732064484115
0.9201572991810932	0.2107610798247199	0.2432931571089844
0.2551647742696570	0.2108549327025644	0.2417630658052984
0.5873459337804641	0.2105278165317134	0.2423050687752265
0.9198509694243794	0.7108920452635178	0.2438818830418353
0.2554832888712230	0.7101233294101784	0.2419395375188660
0.5876733428343976	0.7104378215349283	0.2435501022680302
0.7537744477951747	0.4624839399575532	0.2800739166423005
1.0894147319470595	0.4628109364243860	0.2782432723409649
0.4204873636575015	0.4624617624287893	0.2787811645466054
1.0896160946150533	-0.0373675621973055	0.2780956482103258
0.4197334837935444	-0.0377325491708173	0.2790715024479007
0.7534847145600584	-0.0364668890369441	0.2808701223114878
0.4086245988771988	0.2662223445573109	0.3599059890705774
0.7559610505113660	0.2624557027671251	0.3606607077104723
0.0959279342014111	0.2669201285234888	0.3602322536749690
0.4152041047971187	0.7654327033769955	0.3616492928101805
0.7499408898989396	0.7637664258564471	0.3623679086771504
0.0990423965957562	0.7667146700275259	0.3600477018868199
0.9296566462800119	0.0095053129849849	0.3908385742960452
0.5775370417009817	0.0250775464966872	0.3929630449568021
0.5791580430002298	0.5031451383387742	0.3943429383527478

0.9312938394018636	0.5181085199153455	0.3914331058263313
0.2534539999999978	0.0187629999999999	0.0123030000000028
0.5867879999999985	0.0187629999999999	0.0123030000000028
0.9201210000000017	0.0187629999999999	0.0123030000000028
0.2534539999999978	0.5187629999999999	0.0123030000000028
0.5867879999999985	0.5187629999999999	0.0123030000000028
0.9201210000000017	0.5187629999999999	0.0123030000000028
1.0867859999999965	0.2687629999999999	0.0507869999999997
0.4201189999999997	0.2687629999999999	0.0507869999999997
0.7534530000000004	0.2687629999999999	0.0507869999999997
1.0867859999999965	0.7687629999999999	0.0507869999999997
0.4201189999999997	0.7687629999999999	0.0507869999999997
0.7534530000000004	0.7687629999999999	0.0507869999999997
1.0854756760680855	0.0236037930058804	0.1282065023754152
0.4212969682970076	0.0237137969952520	0.1280054815875171
0.7533267260878488	0.0233927937525299	0.1283659835090674
1.0857406544943342	0.5236129660547022	0.1280601676408298
0.4211006312887804	0.5238431566592064	0.1278683278722098
0.7535574717692860	0.5235633482149940	0.1281536796045777
0.5868978355264282	0.2725662778534602	0.1654254484171551
0.2536158129392454	0.2721949310747948	0.1645375146157197
0.9193103335274627	0.2722090159608275	0.1658849956224659
0.9192432019690279	0.7724320985588575	0.1660316783460270
0.2535889294113322	0.7726134580588297	0.1645148971146788
0.5868929444261231	0.7724166623067967	0.1659202298124776
0.9201081836564359	0.0209249882355437	0.2440605470070578
0.2551473408057203	0.0216628314829343	0.2413430675541235
0.5878384751429477	0.0213317311210090	0.2438478380800313
0.9203321473268165	0.5207802502858362	0.2434741602033937

0.2554267089838133	0.5205511775498207	0.2410944610204288
0.5880910977823003	0.5205757361492374	0.2431326355450163
1.0886735783833568	0.2740131837975879	0.2765306986878112
0.7519245672940683	0.2708015337667072	0.2788980928573292
0.4222081066457266	0.2714196554860978	0.2781418681904185
1.0892868413672609	0.7748554141566345	0.2762220550805176
0.4205100544556228	0.7745607657988455	0.2775734186220317
0.7531156558667335	0.7758133037922799	0.2785422587105987
0.3919156263509949	0.4553060411881424	0.3612313543108755
1.1045591119187133	0.4584457029349322	0.3623815682697994
0.7630891993608544	0.4528926548130063	0.3626841541026285
0.4075946604938202	-0.0375392753532801	0.3632111601448635
0.7486042840199346	-0.0385016790994250	0.3659300105689177
1.1080140475889020	-0.0410829730352572	0.3626352431367289
0.5811057813006674	0.2065851138378039	0.3967296736152672
0.9266112866428653	0.1852132286660794	0.3792956990670447
0.2536039620794369	0.1542520680443110	0.3737040970029016
0.2549600309909767	0.6548398273931005	0.3736997202303449
0.5820288378969917	0.6835535272770403	0.3820884312891855
0.9261419821686028	0.6940405577287719	0.3795574718814932

Optimized XYZ position for the transition states of H_2 heterolysis II on ZnO BCT surface with 0.33 ML OVs

AutoCreatByScript
1.00000000000000
-9.7516200000000008 0.0000000000000000 0.0000000000000000
0.0000000000000000 -10.4606600000000007 0.0000000000000000
0.0000000000000000 0.0000000000000000 24.3835499999999996
H O Zn
2 46 48
Direct
0.5791033374470984 0.5727154730097666 0.4446130526539867
0.5815070853913281 0.6498179881964059 0.4669085903965547
0.2534539999999978 0.2088630000000009 0.0123030000000028
0.5867870000000012 0.2088630000000009 0.0123030000000028
0.9201199999999973 0.2088630000000009 0.0123030000000028
0.2534539999999978 0.7088630000000009 0.0123030000000028
0.5867870000000012 0.7088630000000009 0.0123030000000028
0.9201199999999973 0.7088630000000009 0.0123030000000028
1.0867859999999965 0.4588630000000009 0.0507869999999997
0.4201199999999972 0.4588630000000009 0.0507869999999997
0.7534530000000004 0.4588630000000009 0.0507869999999997
1.0867859999999965 0.9588630000000008 0.0507869999999997
0.4201199999999972 0.9588630000000008 0.0507869999999997
0.7534530000000004 0.9588630000000008 0.0507869999999997
1.0853864569995502 0.2115604370167855 0.1296387268339723
0.4198563078158686 0.2115962288155544 0.1296184330310248
0.7525978491446852 0.2116180388944027 0.1297700921073984
1.0853403668984547 0.7116623427369676 0.1298236718853971

0.4197846626458648	0.7115501113948752	0.1296332073066641
0.7524909797364391	0.7116404560197284	0.1297827616830932
0.5865062869443900	0.4616226489555523	0.1673462517946054
0.2531184562012251	0.4615377399972593	0.1661454477872866
0.9192897221698012	0.4617579103147617	0.1673720780047517
0.9191663634543806	0.9616574704942537	0.1672745640236443
0.2528745977632328	0.9613517619928519	0.1659216429266031
0.5863292944233364	0.9619644751723782	0.1672913366134270
0.9192200708992740	0.2101248761572845	0.2451889894428829
0.2529268829626945	0.2098872411029579	0.2439661503831012
0.5859871677388385	0.2101161161149638	0.2452341613284909
0.9186503231726726	0.7103944437164720	0.2455557632510958
0.2535342265947825	0.7102183351379663	0.2444336923578814
0.5861079217547245	0.7101457070595596	0.2449634195574484
0.7523468349838434	0.4627098070988791	0.2838660643167761
1.0874929397890045	0.4620311403513618	0.2817376636194616
0.4185458474523298	0.4622063617021718	0.2822359693402827
1.0872705727472167	0.9621089691136971	0.2816987160127278
0.4177777614416503	0.9610608610481722	0.2819242325691416
0.7523070809400922	0.9616737302482327	0.2836423250348836
0.4102451956253442	0.2630718299029287	0.3635380515414974
0.7531480935257574	0.2600663171037276	0.3639534789723772
1.0956239321903398	0.2635894566388753	0.3634207562163545
0.4112309471459212	0.7629505635873374	0.3643965081604589
0.7522060350274744	0.7595204599249975	0.3646613007240709
1.0957998606670698	0.7633324866369049	0.3635411482781154
0.9287049021504724	1.0093002253913297	0.3959142321640117
0.5765118789696297	1.0083942026246826	0.3958510889125291
0.5767994008600952	0.5048760021024719	0.3973737761161057

0.9287606132538789	0.5097843551094050	0.3959860568692517
0.2534539999999978	1.0187629999999996	0.0123030000000028
0.5867879999999985	1.0187629999999996	0.0123030000000028
0.9201210000000017	1.0187629999999996	0.0123030000000028
0.2534539999999978	0.5187629999999999	0.0123030000000028
0.5867879999999985	0.5187629999999999	0.0123030000000028
0.9201210000000017	0.5187629999999999	0.0123030000000028
1.0867859999999965	0.2687629999999999	0.0507869999999997
0.4201189999999997	0.2687629999999999	0.0507869999999997
0.7534530000000004	0.2687629999999999	0.0507869999999997
1.0867859999999965	0.7687629999999999	0.0507869999999997
0.4201189999999997	0.7687629999999999	0.0507869999999997
0.7534530000000004	0.7687629999999999	0.0507869999999997
1.0852628401677367	1.0228372000005486	0.1290277811756695
0.4202168257348853	1.0229631902219642	0.1289591424041686
0.7527622049550671	1.0229019362509610	0.1292164006284500
1.0855046192866407	0.5230518063502934	0.1290659310168424
0.4203902482848628	0.5230306827145424	0.1289724575766265
0.7528191025306195	0.5229995378890541	0.1292042227302822
0.5860021270438756	0.2714766295685851	0.1671213847166436
0.2525321538782481	0.2715954833531154	0.1663203297245674
0.9189745046130035	0.2715064145079120	0.1670945706833406
0.9186389342677809	0.7715409254613084	0.1673211273146829
0.2526942742837064	0.7714703262315608	0.1665966723207407
0.5859043316687340	0.7718510957708239	0.1671772914356190
0.9180941454556184	1.0193103706788196	0.2461175398260034
0.2529406264528955	1.0195778286868045	0.2439797235487257
0.5860718048708276	1.0193266728927239	0.2462113413869685
0.9186818946028082	0.5200424945948090	0.2461176253042969

0.2530006402294916	0.5204295166614459	0.2441643511849484
0.5864802806787843	0.5203742918884804	0.2460059150280229
1.0868749978542100	0.2735615689037652	0.2794912852067003
0.7526142537828485	0.2737320045801328	0.2803186228584880
0.4185136578751765	0.2733718271470987	0.2797168623210942
1.0868054214348841	0.7736566654996081	0.2795794245465613
0.4201877349202545	0.7718377036751770	0.2808807783895781
0.7512901663558048	0.7721897575579157	0.2814877071838712
0.3991104887075115	0.4554782473103247	0.3663014016743188
1.1044586727406471	0.4556534186946081	0.3665771421085032
0.7540906345900015	0.4532918264357985	0.3684413646595704
0.3996719914917586	0.9553556968798248	0.3664919241632336
0.7531927975411951	0.9529781611809299	0.3688948416051474
1.1043369671809502	0.9553584051412126	0.3665249112001400
0.5810735303449480	0.1840326419915941	0.3832627916925244
0.9254786350953152	0.1844886807853586	0.3831882603195854
0.2526559971970349	0.1511713533038308	0.3774943669988091
0.2531719086889072	0.6515819259622221	0.3777825961476030
0.5811128234503696	0.7025951372622558	0.3956142805383184
0.9252124567948139	0.6853936720070070	0.3835311289642570

Optimized XYZ position for the transition states of H_2 homolysis on ZnO BCT surface with 0.33 ML OVs

```
AutoCreatByScript
  1.00000000000000
    9.7516200000000008    0.0000000000000000    0.0000000000000000
    0.0000000000000000   10.4606600000000007    0.0000000000000000
    0.0000000000000000    0.0000000000000000   24.3835499999999996
  H    O    Zn
   2    46    48
Direct
  0.2551163323437363  0.5461607916780706  0.4276939438646528
  0.2557155615993442  0.6340903483418172  0.4601527648800196
  0.2534539999999978  0.2088630000000009  0.0123030000000028
  0.5867870000000012  0.2088630000000009  0.0123030000000028
 -0.0798800000000027  0.2088630000000009  0.0123030000000028
  0.2534539999999978  0.7088630000000009  0.0123030000000028
  0.5867870000000012  0.7088630000000009  0.0123030000000028
 -0.0798800000000027  0.7088630000000009  0.0123030000000028
  0.0867859999999965  0.4588630000000009  0.0507869999999997
  0.4201199999999972  0.4588630000000009  0.0507869999999997
  0.7534530000000004  0.4588630000000009  0.0507869999999997
  0.0867859999999965  0.9588630000000008  0.0507869999999997
  0.4201199999999972  0.9588630000000008  0.0507869999999997
  0.7534530000000004  0.9588630000000008  0.0507869999999997
  0.0852606929369736  0.2119685665177982  0.1298286712194827
  0.4200575521839071  0.2119833940251344  0.1298505803594336
  0.7526538457901142  0.2119764422368714  0.1299791278821665
  0.0853648153034255  0.7119581957577588  0.1298130098825504
```

0.4201530027044708	0.7118746830991738	0.1298167923632781
0.7527526391897513	0.7118837772964596	0.1301126912647902
0.5864277238770602	0.4622406101126864	0.1679656835772003
0.2529779325206201	0.4619193448364388	0.1665336702355196
-0.0804480099929410	0.4623357163811397	0.1679380982560950
-0.0806318485822997	0.9621396280346269	0.1677006016812568
0.2530072078169942	0.9620655020315495	0.1664790471881456
0.5865527487680974	0.9621254594265792	0.1677076486556248
-0.0808102202140657	0.2108468517005692	0.2457255317510274
0.2527997866702822	0.2103450396767892	0.2443102466476834
0.5864084791069173	0.2109781845774117	0.2457629869370032
-0.0797971213759372	0.7107786071968938	0.2459760835757626
0.2531974166774362	0.7106529524211311	0.2440590919443973
0.5862070662998521	0.7105900513768261	0.2459280335507524
0.7530686526461542	0.4631179693455286	0.2845896921163324
0.0884415961246551	0.4629230049352096	0.2830303125288302
0.4174696640978792	0.4628535820887298	0.2830829766601522
0.0874948740898039	0.9616336069746936	0.2825069278219902
0.4185018683433588	0.9617402987062141	0.2825241538683275
0.7528563258473276	0.9630432410470819	0.2844332781462259
0.4111391370495080	0.2601075206019566	0.3636997311552695
0.7537215044923924	0.2605843617625956	0.3650928220320514
0.0956909445354628	0.2605524229735968	0.3636029181813539
0.4139975033096218	0.7636303034866568	0.3640963580384364
0.7538462074256699	0.7595377780935453	0.3646248184800363
0.0941085801040503	0.7636367164763707	0.3642292189897905
-0.0714189318103308	1.0089989870679679	0.3974213007371037
0.5789398061523298	1.0094372910425955	0.3976306065889210
0.5746404602549287	0.5070682662874804	0.3961062815125130

-0.0671210895606913	0.5078261950333495	0.3963390770863394
0.2534539999999978	1.0187629999999996	0.0123030000000028
0.5867879999999985	1.0187629999999996	0.0123030000000028
-0.0798789999999983	1.0187629999999996	0.0123030000000028
0.2534539999999978	0.5187629999999999	0.0123030000000028
0.5867879999999985	0.5187629999999999	0.0123030000000028
-0.0798789999999983	0.5187629999999999	0.0123030000000028
0.0867859999999965	0.2687629999999999	0.0507869999999997
0.4201189999999997	0.2687629999999999	0.0507869999999997
0.7534530000000004	0.2687629999999999	0.0507869999999997
0.0867859999999965	0.7687629999999999	0.0507869999999997
0.4201189999999997	0.7687629999999999	0.0507869999999997
0.7534530000000004	0.7687629999999999	0.0507869999999997
0.0854985450598456	1.0231579977615903	0.1292080018114565
0.4203801836711091	1.0232449341688807	0.1291934663885240
0.7529068075269129	1.0230529227607004	0.1293518529524167
0.0855308943570137	0.5231595401235052	0.1292316218615305
0.4203449417486883	0.5230987620939560	0.1292493281222655
0.7528693749772504	0.5229765906129195	0.1295375012075711
0.5862306878305474	0.2719413577732151	0.1675395532066430
0.2524778779642972	0.2718810725484692	0.1666071679165188
-0.0810652482812534	0.2720368187519169	0.1675144479521900
-0.0807493868785819	0.7718152739826576	0.1676408512873145
0.2526357080322874	0.7720658324597668	0.1664717897722260
0.5861007585336325	0.7717750031926297	0.1676272160442408
-0.0811288314996633	1.0201573462114304	0.2465707561553897
0.2528646509469779	1.0202838633906957	0.2446925038468493
0.5867565337240023	1.0203760654706768	0.2465536045491544
-0.0806998025635215	0.5202016738948427	0.2469602306131731

0.2529124459562541	0.5209940604296796	0.2447385836429487
0.5864601340336021	0.5199803754639083	0.2470071896673660
0.0869139523815143	0.2739775037167978	0.2801475868690035
0.7527935062067891	0.2742763929701827	0.2813843887218641
0.4185058366425660	0.2739428410748537	0.2801547391051238
0.0887228333777525	0.7724755702104009	0.2805524663581629
0.4178079263454531	0.7726221957772883	0.2804377282867562
0.7531109715355758	0.7745379015315822	0.2807850561616888
0.3963076900082395	0.4532051771260972	0.3673316993908542
0.1109970809929832	0.4537233054732763	0.3673229260407562
0.7541126105637017	0.4525916704066672	0.3702944387324982
0.4049428442420596	0.9567228007779089	0.3670053002180570
0.7535880596316884	0.9521944592926109	0.3703125949115190
0.1031138835954332	0.9566188987560086	0.3669520777445619
0.5820542182962916	0.1846963046310346	0.3845780330280724
-0.0746809965182458	0.1841574273885484	0.3842199256590773
0.2532447905658011	0.1499562821138209	0.3781466683942165
0.2540732434790863	0.6838418784802958	0.3947544227548502
0.5832016269634952	0.6818288391508656	0.3837539649859411
-0.0752546989632482	0.6826950139165896	0.3840581080313667

Optimized XYZ position for the transition states of hetero_H_I on ZnO BCT surface with 0.33 ML OVs

```
AutoCreatByScript
  1.00000000000000
    9.7516200000000008    0.0000000000000000    0.0000000000000000
    0.0000000000000000   10.4606600000000007    0.0000000000000000
    0.0000000000000000    0.0000000000000000   24.3835499999999996
  H    C    O    Zn
    2    1    47    48
Direct
  0.5790278790326717  0.4955339135302416  0.4360159732500831
  0.4956043176292274  0.2350278158745643  0.4604508251010595
  0.3370488384287349  0.2132964546327556  0.4734741092679433
  0.2534539999999978  0.2088630000000009  0.0123030000000028
  0.5867870000000012  0.2088630000000009  0.0123030000000028
  0.9201199999999973  0.2088630000000009  0.0123030000000028
  0.2534539999999978  0.7088630000000009  0.0123030000000028
  0.5867870000000012  0.7088630000000009  0.0123030000000028
  0.9201199999999973  0.7088630000000009  0.0123030000000028
  0.0867859999999965  0.4588630000000009  0.0507869999999997
  0.4201199999999972  0.4588630000000009  0.0507869999999997
  0.7534530000000004  0.4588630000000009  0.0507869999999997
  0.0867859999999965 -0.0411369999999992  0.0507869999999997
  0.4201199999999972 -0.0411369999999992  0.0507869999999997
  0.7534530000000004 -0.0411369999999992  0.0507869999999997
  0.0851816787010465  0.2125637467085149  0.1296695129208729
  0.4206738767722969  0.2124915849812524  0.1294051013419129
  0.7546490500260505  0.2123373161408430  0.1297577308087653
```

0.0857560936768170	0.7118592626560893	0.1294417040334564
0.4206803909914679	0.7119043610747537	0.1295800574375910
0.7537157717115862	0.7120411818767929	0.1298268238839108
0.5875764821400663	0.4627908281190776	0.1669146704908423
0.2532054016172741	0.4626549414923076	0.1655123609253218
0.9198611953221488	0.4624622811587595	0.1675689955697960
0.9203192518410234	-0.0373426453956355	0.1670102415513132
0.2533347925834610	-0.0378697947428449	0.1649296946346048
0.5871776060857524	-0.0378486292033046	0.1664805016658021
0.9210893829495758	0.2138923601539889	0.2468795815010243
0.2538150990716857	0.2129063142002009	0.2429045792565052
0.5889507257655148	0.2115731070053546	0.2437494797657200
0.9227965506714577	0.7101522500585042	0.2450561718102207
0.2534901173952482	0.7105660606864684	0.2433227806863036
0.5885635697180536	0.7113971396905174	0.2451812018453642
0.7536967273529193	0.4631032256546473	0.2843113752884474
0.0894519012059657	0.4623607890719748	0.2827131893047272
0.4204445261382743	0.4645576370767708	0.2808588494480944
0.0904806951697774	-0.0347636992691230	0.2809397464412446
0.4185666778967741	-0.0366828405349290	0.2796051820866572
0.7553698630524507	-0.0355375521657413	0.2813062402538234
0.4147425938830396	0.2668136434989707	0.3593674604692409
0.7511997169458309	0.2626361907968053	0.3666453920610670
0.0970266674864584	0.2664193838861584	0.3659343466710605
0.4101698842854383	-0.2346179620158849	0.3620811225502202
0.7580836163198842	-0.2364662069177479	0.3627502902542113
0.1016625481431639	-0.2321834961813203	0.3628481664439453
0.9337617058794735	0.0100728859735500	0.3927212238071790
0.5779466275918378	0.0116759470369143	0.3909677054046691

0.5827275302420448	0.5190401233716685	0.3972427345293958
0.9317285065752745	0.5185102984785164	0.3979097222057200
0.3155434014823615	0.2244668503158142	0.5206303362522937
0.2534539999999978	0.0187629999999999	0.0123030000000028
0.5867879999999985	0.0187629999999999	0.0123030000000028
0.9201210000000017	0.0187629999999999	0.0123030000000028
0.2534539999999978	0.5187629999999999	0.0123030000000028
0.5867879999999985	0.5187629999999999	0.0123030000000028
0.9201210000000017	0.5187629999999999	0.0123030000000028
0.0867859999999965	0.2687629999999999	0.0507869999999997
0.4201189999999997	0.2687629999999999	0.0507869999999997
0.7534530000000004	0.2687629999999999	0.0507869999999997
0.0867859999999965	-0.2312370000000001	0.0507869999999997
0.4201189999999997	-0.2312370000000001	0.0507869999999997
0.7534530000000004	-0.2312370000000001	0.0507869999999997
0.0856016593200272	0.0240755228626474	0.1285339159801509
0.4211134386538776	0.0239118659827559	0.1286220373020937
0.7537910760148115	0.0236452163211155	0.1289964803036787
0.0853813737358356	0.5234754264671362	0.1287077004336677
0.4209481179844354	0.5235253574143557	0.1287048377412659
0.7540595035851189	0.5234805551316913	0.1289261528802546
0.5874863039217412	0.2724873257834340	0.1664359000662940
0.2528424736401521	0.2728534570075454	0.1658036688221435
0.9198884127928699	0.2721610729938678	0.1681676784049585
0.9200611190683198	-0.2274433166324199	0.1669870453852565
0.2529417671048194	-0.2274760040550727	0.1657792840887874
0.5867842762681570	-0.2275344259418914	0.1667387021067896
0.9226402788802395	0.0235019040927370	0.2454580306608467
0.2538159720671311	0.0237467207081984	0.2424546079954620

0.5876853456920759	0.0217147103012523	0.2451669779927926
0.9216912758222644	0.5195408050820375	0.2460325289968343
0.2532172095113618	0.5207182627877146	0.2431967376009853
0.5887704830552150	0.5210958569021645	0.2450496428461465
0.0918767144930757	0.2717764100651508	0.2822655785300033
0.7507522418002787	0.2704267369052902	0.2827468820486012
0.4216511779103984	0.2700784875209890	0.2793393803880797
0.0894337674156272	-0.2221976078075531	0.2791980643255432
0.4190158624630039	-0.2238405370822074	0.2779804177984971
0.7566058211973735	-0.2229997917551509	0.2785871785810323
0.3858510626859167	0.4512286987551898	0.3619891209304361
0.1031922844555620	0.4567194379004704	0.3671337352847466
0.7691665861237519	0.4511502239674731	0.3660755081210972
0.3981557645071966	-0.0333125813839609	0.3637497245723270
0.7550577963040357	-0.0379844652329248	0.3659098028347130
0.1168745201324566	-0.0349407091163034	0.3659165014632912
0.9257051930862418	0.1863524893239438	0.3837878233936323
0.2545813349567518	0.6534964198404315	0.3761382796539539
0.5833857249670144	0.7058891704377754	0.3809716132435810
0.9314970210615326	0.6939910277742605	0.3832099778405463
0.5769783573376867	0.1936759241871721	0.4007683146053617
0.2611339587279847	0.1667354093965640	0.3939841328363660

Optimized XYZ position for the transition states of hetero_H_II on ZnO BCT surface with 0.33 ML OVs

```
AutoCreatByScript
  1.00000000000000
    9.7516200000000008    0.0000000000000000    0.0000000000000000
    0.0000000000000000   10.4606600000000007    0.0000000000000000
    0.0000000000000000    0.0000000000000000   24.3835499999999996
  H    C    O    Zn
    1     1    47    48
Direct
  0.5826473256167061  0.4202546017169462  0.4421031258400992
  0.5865647938119265  0.3155895659154176  0.4669586025070336
  0.2534539999999978  0.2088630000000009  0.0123030000000028
  0.5867870000000012  0.2088630000000009  0.0123030000000028
  0.9201199999999973  0.2088630000000009  0.0123030000000028
  0.2534539999999978  0.7088630000000009  0.0123030000000028
  0.5867870000000012  0.7088630000000009  0.0123030000000028
  0.9201199999999973  0.7088630000000009  0.0123030000000028
  1.0867859999999965  0.4588630000000009  0.0507869999999997
  0.4201199999999972  0.4588630000000009  0.0507869999999997
  0.7534530000000004  0.4588630000000009  0.0507869999999997
  1.0867859999999965 -0.0411369999999992  0.0507869999999997
  0.4201199999999972 -0.0411369999999992  0.0507869999999997
  0.7534530000000004 -0.0411369999999992  0.0507869999999997
  1.0858059258853094  0.2122219455374554  0.1294473764534415
  0.4200611785220781  0.2118980687723211  0.1291572487510096
  0.7533446314960973  0.2118478014529201  0.1293506261853130
  1.0856074100998583  0.7121305791977338  0.1294688328938073
```

0.4200804909973805	0.7121532675483535	0.1294563169309488
0.7530006224245847	0.7121545241923092	0.1296410599946315
0.2532989164260279	0.4622557611746070	0.1651130937270208
0.2530305980503729	-0.0378166800074288	0.1650589296148727
0.5868663519314228	0.4623746468931158	0.1661388577500474
0.9197233186191910	0.4624168003220669	0.1665392309028669
0.9196951996666656	-0.0376308905726440	0.1665768789719433
0.5866352811092095	-0.0379347390790502	0.1664378773804626
0.2541674017159245	0.2116659669756026	0.2431953706235837
0.2531704474891615	0.7106553947505971	0.2432679543362034
0.5865134540331969	0.2106237712777996	0.2432803975744226
0.9198530927561560	0.2116133241172732	0.2444177746878665
0.5866731117346775	0.7106596477984171	0.2444745150404650
0.9203086174262597	0.7110563042553940	0.2446403695994502
0.4193973904622978	0.4624334559238571	0.2803984154183849
1.0883330653024701	0.4635374367370285	0.2807241842722589
0.4179615018042317	-0.0377739026957179	0.2800797315978273
1.0883937673659374	-0.0365893335485753	0.2805748805160491
0.7529291343013923	0.4627642324273649	0.2817642569777775
0.7533180591710636	-0.0368674654561721	0.2817638375234395
0.4116155898057032	0.2645301244581058	0.3621435448126410
0.0959499220469727	0.2650816441412327	0.3620887035000300
0.4118659005178687	0.7635810939541161	0.3624781279317914
0.0973951404207593	0.7649810827201338	0.3626059281198946
0.7532023579778305	0.2606401256175964	0.3621422792190001
0.7531680581827372	0.7613530243428834	0.3628642317960908
0.9292664202599219	0.0095851198595468	0.3934684515796392
0.5774071546149406	0.0120974715777993	0.3932389800883192
0.9292454702691529	0.5128596489133795	0.3945697353552688

0.5791451317832487	0.5093414928429573	0.3960470436928753
0.5926175541318128	0.2989837719912936	0.5152099572974081
0.2534539999999978	0.0187629999999999	0.0123030000000028
0.5867879999999985	0.0187629999999999	0.0123030000000028
0.9201210000000017	0.0187629999999999	0.0123030000000028
0.2534539999999978	0.5187629999999999	0.0123030000000028
0.5867879999999985	0.5187629999999999	0.0123030000000028
0.9201210000000017	0.5187629999999999	0.0123030000000028
1.0867859999999965	0.2687629999999999	0.0507869999999997
0.4201189999999997	0.2687629999999999	0.0507869999999997
0.7534530000000004	0.2687629999999999	0.0507869999999997
1.0867859999999965	0.7687629999999999	0.0507869999999997
0.4201189999999997	0.7687629999999999	0.0507869999999997
0.7534530000000004	0.7687629999999999	0.0507869999999997
0.7530703809115878	0.0233501731061516	0.1288338992299918
1.0853509830433363	0.0238536279226331	0.1284735033369638
0.4206767662382236	0.0235868053147678	0.1284830139975623
0.7535358329188021	0.5237725831039275	0.1286523595628600
1.0855600793603359	0.5237842997481852	0.1284760178029078
0.4206278084713156	0.5239165564802866	0.1283652077174713
0.2532334108605587	0.2725574608961717	0.1656742756419101
0.2527349662790278	0.7727134080373286	0.1657384437364523
0.5864290212451659	0.2724085095033197	0.1660001454828761
0.9192321692617933	0.2724222324586051	0.1665694819175251
0.9193062813836052	0.7726292618894658	0.1666584753203803
0.5862428895567121	0.7725139450853845	0.1665423459551812
0.2528540938282275	0.0220760683603475	0.2426157224818814
0.2535111746543519	0.5210151376039190	0.2427026999120244
0.9198371469762673	0.0214122134147751	0.2451111609868516

0.5865913087712583	0.0209047102740585	0.2450666831596543
0.9199074134314740	0.5209330853382506	0.2448984823016946
0.5870676676374494	0.5206219107660662	0.2443405967412545
0.4216232072368783	0.2716305720335881	0.2795634560416386
1.0878377840743834	0.2748847274010101	0.2783773397802209
0.4190172343633378	0.7743340710081532	0.2784817940675655
1.0877335648732689	0.7754175487923172	0.2786718338420209
0.7511942727990776	0.2715004763655807	0.2798183036732571
0.7532975434114341	0.7750679338783280	0.2791111798576677
0.3964456094364376	0.4550189268363598	0.3632036222658135
0.1041783759815501	0.4562891959814586	0.3653817674876271
0.4035643595767582	-0.0414312070886221	0.3643890122109010
0.1068723823637052	-0.0427098504990217	0.3655739323008012
0.7591454257561441	0.4523609891302983	0.3646815984946170
0.7518941863674214	-0.0430339157013818	0.3667114103625810
0.2543304015069266	0.1533080758736216	0.3755050818679653
0.2535620317192010	0.6523455369880038	0.3759075033115004
0.9253330068860580	0.1848739549243947	0.3806428886517137
0.5816408169733280	0.1920419926468314	0.3968851327958178
0.9263411761920473	0.6885361786718893	0.3822379130445656
0.5820560413355171	0.6883446395490441	0.3828032833753887

Optimized XYZ position for the transition states of homo_H_I on ZnO BCT surface with 0.33 ML OVs

```
AutoCreatByScript
  1.00000000000000
    9.7516200000000008    0.0000000000000000    0.0000000000000000
    0.0000000000000000   10.4606600000000007    0.0000000000000000
    0.0000000000000000    0.0000000000000000   24.3835499999999996
  H    C    O    Zn
   2     1    47    48
Direct
  0.2461797462789209  0.5300059525018957  0.3845837371816745
  0.3423827948963002  0.7109885245841364  0.4622845356177430
  0.4883821585491430  0.7218366014297000  0.4772142895020331
  0.2534539999999978  1.2088630000000009  0.0123030000000028
  0.5867870000000012  1.2088630000000009  0.0123030000000028
 -0.0798800000000027  1.2088630000000009  0.0123030000000028
  0.2534539999999978  0.7088630000000009  0.0123030000000028
  0.5867870000000012  0.7088630000000009  0.0123030000000028
 -0.0798800000000027  0.7088630000000009  0.0123030000000028
  0.0867859999999965  0.4588630000000009  0.0507869999999997
  0.4201199999999972  0.4588630000000009  0.0507869999999997
  0.7534530000000004  0.4588630000000009  0.0507869999999997
  0.0867859999999965  0.9588630000000008  0.0507869999999997
  0.4201199999999972  0.9588630000000008  0.0507869999999997
  0.7534530000000004  0.9588630000000008  0.0507869999999997
  0.4201272811435670  0.2116818355282426  0.1294051087895483
  0.0852779032183562  0.2116883353928566  0.1294193412533009
  0.7528732516545802  0.2117136188850244  0.1296032101092708
```

0.4200964493804401	0.7116569591794192	0.1293034882610462
0.0852901526251191	0.7118697506221381	0.1295816883080111
0.7533178756826295	0.7118133912800307	0.1297752515757873
0.2528358017956280	0.4614292359326455	0.1652220589013882
0.2530114418484520	0.9618368581809481	0.1652007150348005
0.5863106828591047	0.4618002266764630	0.1667652863762601
-0.0802896127294052	0.4620788077625291	0.1668871972130003
-0.0803534692898497	0.9618732670208080	0.1667778146909738
0.5867885009533196	0.9620639681506874	0.1664989663166377
0.2527068904198485	0.2100686228089959	0.2430580640620301
0.2531006690660553	0.7107032001751731	0.2430102907770554
0.5866062725238944	0.2105897465241232	0.2445454412266019
-0.0800968604676885	0.2101381968329417	0.2446324991900699
0.5862743640810744	0.7106121814164820	0.2440189340924024
-0.0794755342717284	0.7116871375645109	0.2460388416749209
0.4163935350011829	0.4625189034061079	0.2804461032031647
0.4187534163503313	0.9625079098054774	0.2800914809287527
0.0882088262410541	0.4629059187157333	0.2811197560438365
0.0883151047140491	0.9618382105041519	0.2810977350375032
0.7530153011749178	0.4630523040839806	0.2819878021221030
0.7532583810749560	0.9620024454627003	0.2826668649073903
0.4104913772028999	0.2655709587561395	0.3622179553754696
0.0954868294620807	0.2649360939798629	0.3624658000726864
0.7534984330393532	0.2643882195862884	0.3629993630642852
0.7481386808973723	0.7610324782172850	0.3646446329109321
0.4140756711251254	0.7659867487558776	0.3602779763266848
0.0933407025777740	0.7675754118633811	0.3650756335430783
-0.0742179380280896	0.5145215704053855	0.3950652743473134
0.5778693201251213	0.5154735469301401	0.3942962896463060

-0.0726757119893826	1.0121699713834671	0.3952453756077061
0.5785634226686270	1.0128171067131009	0.3951512115266061
0.5010011019080821	0.7258676394563749	0.5250892667174469
0.2534539999999978	1.0187629999999996	0.0123030000000028
0.5867879999999985	1.0187629999999996	0.0123030000000028
-0.0798789999999983	1.0187629999999996	0.0123030000000028
0.2534539999999978	0.5187629999999999	0.0123030000000028
0.5867879999999985	0.5187629999999999	0.0123030000000028
-0.0798789999999983	0.5187629999999999	0.0123030000000028
0.0867859999999965	0.2687629999999999	0.0507869999999997
0.4201189999999997	0.2687629999999999	0.0507869999999997
0.7534530000000004	0.2687629999999999	0.0507869999999997
0.0867859999999965	0.7687629999999999	0.0507869999999997
0.4201189999999997	0.7687629999999999	0.0507869999999997
0.7534530000000004	0.7687629999999999	0.0507869999999997
0.7531782898789334	1.0229830076091500	0.1288891583851911
0.4206488187583750	1.0231734709714602	0.1286382861936825
0.0853900084260026	1.0231719162545170	0.1286272012769364
0.4205635154444261	0.5234186988201135	0.1285532824098650
0.0852469711858157	0.5236249892668410	0.1284926524103578
0.7528585358451275	0.5233916777715190	0.1289709447482041
0.2526333378827680	0.2718458152636276	0.1655243164748179
0.2529500745608240	0.7722981563581113	0.1657800726324427
0.5862861545753606	0.2719522871032480	0.1665955714524482
-0.0809991911266169	0.2719640908264753	0.1666837840662918
0.5861764926614043	0.7721343594036091	0.1667609179275185
-0.0806904240077682	0.7719252880031104	0.1674189191459769
0.2520537702966704	0.5222954467754091	0.2423617343727682
0.2535189603888159	1.0196620017135747	0.2430244505581609

-0.0808592462495705	1.0190750922238223	0.2454276487451108
0.5874796143311527	1.0195343808262054	0.2451801816043474
0.5866123899731742	0.5216007279562158	0.2451668572189948
-0.0810050827452164	0.5218484022139772	0.2451251729710830
0.4185137618101177	0.2727272131538644	0.2783327241366907
0.0874037160988110	0.2731272364795108	0.2784823000941337
0.7531778699456685	0.2743984263837240	0.2794874187923185
0.0903829152500482	0.7728341099434596	0.2804035263289182
0.7503861596758289	0.7720828472192789	0.2813697170046379
0.4192685256969378	0.7706822854216450	0.2800805119570690
0.4142527762059300	0.4584129272596810	0.3622740747478458
0.0916951106074593	0.4557301677939895	0.3634139016570546
0.4064995732366053	0.9570071544713002	0.3638370099612811
0.1020601494364714	0.9604858853959225	0.3652215090219044
0.7501600262641384	0.4583782710833605	0.3669090349127783
0.7528144791171180	0.9529785353072994	0.3679776775297043
0.2541802322711135	1.1524131376160407	0.3762066462101620
0.5821721914281497	1.1877440922306888	0.3825463048346555
-0.0757638367517536	1.1870123840728848	0.3826274763348241
-0.0788605843416863	0.6906469945754208	0.3833562010786030
0.5782061855556053	0.6987482433111859	0.4000183325125478
0.2563310401585775	0.7060221704395246	0.4017218740468868

Optimized XYZ position for the transition states of homo_H_II on ZnO BCT surface with 0.33 ML OVs

```
AutoCreatByScript
  1.00000000000000
    9.7516200000000008    0.0000000000000000    0.0000000000000000
    0.0000000000000000   10.4606600000000007    0.0000000000000000
    0.0000000000000000    0.0000000000000000   24.3835499999999996
  H    C    O    Zn
    1     1    47    48
Direct
  0.2550403429214724  0.4918009432490010  0.4031998883352135
  0.2544255499503207  0.5715995477746958  0.4497266737993936
  0.2534539999999978  0.2088630000000009  0.0123030000000028
  0.5867870000000012  0.2088630000000009  0.0123030000000028
 -0.0798800000000027  0.2088630000000009  0.0123030000000028
  0.2534539999999978  0.7088630000000009  0.0123030000000028
  0.5867870000000012  0.7088630000000009  0.0123030000000028
 -0.0798800000000027  0.7088630000000009  0.0123030000000028
  0.0867859999999965  0.4588630000000009  0.0507869999999997
  0.4201199999999972  0.4588630000000009  0.0507869999999997
  0.7534530000000004  0.4588630000000009  0.0507869999999997
  0.0867859999999965  0.9588630000000008  0.0507869999999997
  0.4201199999999972  0.9588630000000008  0.0507869999999997
  0.7534530000000004  0.9588630000000008  0.0507869999999997
  0.4204889967131801  0.2110587248841202  0.1297334805857321
  0.0852504234002580  0.2110560990729091  0.1297481356587263
  0.7528981591238220  0.2110331176293416  0.1298953065526999
  0.0854793046443515  0.7113572762518250  0.1299357412715406
```

0.4203165312361831	0.7111506991941214	0.1299229290597957
0.7528631044373976	0.7110943365666926	0.1302594891765898
0.2531176882069376	0.4612366583638611	0.1658730502113471
0.2531920179294669	0.9613180873524833	0.1655145569920524
-0.0800738053067175	0.4616827986861417	0.1674594825369828
0.5865236731611587	0.4616967350205056	0.1674166015411823
-0.0804478464330777	0.9615701769018427	0.1671256108653632
0.5866602816505222	0.9614919905700464	0.1670780722714961
0.2534341734875102	0.2097648393011009	0.2429555553457658
0.2537719250486665	0.7107343368999053	0.2438333980823573
-0.0804726388976278	0.2107540937878758	0.2448906509534763
0.5874944765603450	0.2105570014560099	0.2447324177382201
-0.0788014842244140	0.7110117395148132	0.2456809495340169
0.5857559020775061	0.7108913030815397	0.2457145078820786
0.0897035843646434	0.4628019117362600	0.2821239584050455
0.4182567224927026	0.4625422100202993	0.2822762879559104
0.0881929981453859	0.9630340540453186	0.2810575856239250
0.4190426981644269	0.9632156894701827	0.2813076583009943
0.7538972129803928	0.4641712082189010	0.2837369494087717
0.7533870470656074	0.9639829746962514	0.2830703641307769
0.0953057756077826	0.2596433846147840	0.3621561070955663
0.4144451089376185	0.2586882011349353	0.3618533956895384
0.7546177926900509	0.2644632750430542	0.3636857903996625
0.7542775253454134	0.7636393957229525	0.3632970847212672
0.0928843191803479	0.7686982995192495	0.3640280355823951
0.4152315161665815	0.7685869933568422	0.3641750649544879
-0.0724451901121494	0.0108673964410097	0.3965879545337846
0.5806013330023750	0.0101456806264125	0.3969531076554498
-0.0719383432013520	0.5146534475075422	0.3985568268537886

0.5823161743588476	0.5145927421440375	0.3981578990324521
0.2507734806939389	0.5155386673795199	0.4920334895297325
0.2534539999999978	0.0187629999999999	0.0123030000000028
0.5867879999999985	0.0187629999999999	0.0123030000000028
-0.0798789999999983	0.0187629999999999	0.0123030000000028
0.2534539999999978	0.5187629999999999	0.0123030000000028
0.5867879999999985	0.5187629999999999	0.0123030000000028
-0.0798789999999983	0.5187629999999999	0.0123030000000028
0.0867859999999965	0.2687629999999999	0.0507869999999997
0.4201189999999997	0.2687629999999999	0.0507869999999997
0.7534530000000004	0.2687629999999999	0.0507869999999997
0.0867859999999965	0.7687629999999999	0.0507869999999997
0.4201189999999997	0.7687629999999999	0.0507869999999997
0.7534530000000004	0.7687629999999999	0.0507869999999997
0.4208227498578560	0.0225684156776109	0.1280887464627649
0.0852249940040381	0.0226036337600135	0.1281528283481501
0.0853455404381820	0.5231181428905392	0.1280711490968977
0.4206422086263250	0.5228576714668997	0.1280236882095125
0.7531170816483163	0.0223389227666352	0.1283872855695348
0.7532114053644949	0.5225226015517561	0.1284211108118597
0.2527683162290930	0.2716214207655557	0.1647632001905549
-0.0810785066216242	0.2717069968192318	0.1662797181487823
0.5866260540556198	0.2717725671535064	0.1661811437967575
0.2527960863986166	0.7719745581721272	0.1653803418512529
-0.0807978626330577	0.7718210456924456	0.1666800353171715
0.5862124191121634	0.7717594788166757	0.1666744973813875
0.2537065577113227	0.0199666802884014	0.2423325977535551
0.2541680492915767	0.5220539558496089	0.2424406845765245
-0.0810284398980340	0.0203352719106386	0.2447475627213391

0.5878067104699705	0.0202077863491517	0.2446502467560077
-0.0804989706818635	0.5214771903696257	0.2447795130600382
0.5881153796319621	0.5214765744487547	0.2447687707276638
0.0880212750605295	0.2718814972596423	0.2773990305454549
0.4189994750820027	0.2716933885097589	0.2772157933292791
0.7532449611282174	0.2750105950705631	0.2793183855736857
0.7534103709834181	0.7752563645999987	0.2792595745598656
0.0894268109432007	0.7737849651811984	0.2793581924224420
0.4179066005889108	0.7742364357018431	0.2793878284018096
0.4268073311696820	0.4461453585222137	0.3628669698512842
0.0835196751887222	0.4472956949850457	0.3626861442740260
0.4078908879652690	0.9628906148935545	0.3642429936701423
0.0999823695557540	0.9632277628579290	0.3637802787261368
0.7555015625479079	0.4561452514653803	0.3679395380870864
0.7540063486757506	0.9554892453534507	0.3673795977368606
0.2545216735718875	0.1518223913217032	0.3779790967990080
-0.0763995601733685	0.1851947713428703	0.3836125422521026
0.5862487899623872	0.1842459512237634	0.3835025099571838
-0.0764621084371817	0.6897059425313921	0.3844183676669357
0.5847757784610811	0.6898666690735724	0.3842589425362180
0.2539162326089290	0.7237201220325835	0.4026261684083566

Optimized XYZ position for the transition states of C-O breakage on ZnO BCT surface with 0.33 ML OVs

AutoCreatByScript
1.00000000000000
9.7516200000000008 0.0000000000000000 0.0000000000000000
0.0000000000000000 10.4606600000000007 0.0000000000000000
0.0000000000000000 0.0000000000000000 24.3835499999999996
C O Zn
1 47 48
Direct
0.2557250261111038 0.5085513657460649 0.4050174204045364
0.2534539999999978 0.2088630000000009 0.0123030000000028
0.5867870000000012 0.2088630000000009 0.0123030000000028
-0.0798800000000027 0.2088630000000009 0.0123030000000028
0.2534539999999978 0.7088630000000009 0.0123030000000028
0.5867870000000012 0.7088630000000009 0.0123030000000028
-0.0798800000000027 0.7088630000000009 0.0123030000000028
0.0867859999999965 0.4588630000000009 0.0507869999999997
0.4201199999999972 0.4588630000000009 0.0507869999999997
0.7534530000000004 0.4588630000000009 0.0507869999999997
0.0867859999999965 0.9588630000000008 0.0507869999999997
0.4201199999999972 0.9588630000000008 0.0507869999999997
0.7534530000000004 0.9588630000000008 0.0507869999999997
0.4250375658839755 0.2122028578341749 0.1289125869310545
0.7529534246951642 0.2120820594280233 0.1289908684291924
0.0879276226354109 0.2132826664743434 0.1292018784286896
0.4206729019254768 0.7129337662314394 0.1296288993577878
0.7526422620708005 0.7130084748322484 0.1300112004295978

0.0860738057012690	0.7129362146885951	0.1296273744727758
0.2546338887011104	0.9626093993417327	0.1654198128580819
0.2544503275768143	0.4647564745974341	0.1663201854288427
-0.0799379795914562	0.4646107783286621	0.1668761906929998
-0.0790132411640950	0.9631631359629663	0.1666442782905010
0.5868039361416796	0.4636659644795793	0.1676192028078873
0.5877278657611620	0.9628913458216701	0.1675787163422791
0.2605915890101725	0.2125209506533718	0.2397091965522887
-0.0805962510792221	0.2130059839007824	0.2413536305046348
0.2544014482988960	0.7135662030093416	0.2441518363361662
0.5850429708568289	0.7127622562849768	0.2460031304651364
-0.0791027439186941	0.7146360832582469	0.2453026251998717
0.5920212821696912	0.2130419832301100	0.2453767513001378
0.0912608612554749	0.9675815461999270	0.2811931665100460
0.4199713720738151	0.9669863451485280	0.2823595992615852
0.0889166586104217	0.4664043316915101	0.2816043158877514
-0.2433530192798658	0.9671184067419699	0.2831210638554895
0.4196310971483281	0.4615859979159084	0.2837954320821189
0.7539647919306610	0.4637566177307377	0.2825487127251696
0.0879148465696436	0.2456216568330168	0.3468813788516745
0.7523867619187697	0.7658826431562504	0.3630067421698080
0.0907526238129382	0.7777096291553551	0.3633646539866176
0.4081605552484546	0.7716500321216836	0.3652938192050036
0.4222869740313733	0.2502875909081971	0.3612025128314343
-0.2381544110929026	0.2649559524248447	0.3655383380541019
-0.0761748617550561	0.5171359355550428	0.3935784967844944
0.5817993878999435	1.0082432404677706	0.3964674036824407
0.5816661307662566	0.5170805482999566	0.3955421616367151
-0.0698797892632214	0.0156674572468478	0.3987079767204486

0.1426743271067633	0.2960581068785509	0.4339359861579506
0.2534539999999978	0.0187629999999999	0.0123030000000028
0.5867879999999985	0.0187629999999999	0.0123030000000028
-0.0798789999999983	0.0187629999999999	0.0123030000000028
0.2534539999999978	0.5187629999999999	0.0123030000000028
0.5867879999999985	0.5187629999999999	0.0123030000000028
-0.0798789999999983	0.5187629999999999	0.0123030000000028
0.0867859999999965	0.2687629999999999	0.0507869999999997
0.4201189999999997	0.2687629999999999	0.0507869999999997
0.7534530000000004	0.2687629999999999	0.0507869999999997
0.0867859999999965	0.7687629999999999	0.0507869999999997
0.4201189999999997	0.7687629999999999	0.0507869999999997
0.7534530000000004	0.7687629999999999	0.0507869999999997
0.4224506449186747	0.0237683464107153	0.1286878548845686
0.7537668449743254	0.0234815437714407	0.1289794352086502
0.0870854662261964	0.0244450535877518	0.1286731238906339
0.7527416353108158	0.5244773072067207	0.1290545066328873
0.4217463634510866	0.5247459724086705	0.1287281746733566
0.0866833696271647	0.5246565547095249	0.1288899070048630
0.2578941689952514	0.2753818499990266	0.1632414800814238
-0.0809415223638995	0.2747772220132446	0.1645071768508221
0.2531980837334082	0.7728724659102507	0.1661594082949234
0.5861015313920274	0.7727957283176951	0.1674399119947558
-0.0803495635871826	0.7733070879516352	0.1668672310926310
0.5892490740732284	0.2735008722038993	0.1676620531821044
0.2561547986271381	0.0239972582399715	0.2428518524276814
-0.0777166398230793	0.0237971455563114	0.2450622333186585
0.5896653013133387	0.0233885826554075	0.2459187822998802
0.2567102582104147	0.5236275817588183	0.2451544826588641

-0.0802274758162551	0.5242131821622575	0.2457495392804968
0.5859174975062148	0.5214243997094260	0.2465912516485124
0.0914477294672127	0.2788579969884878	0.2690834732875255
0.0893209460955654	0.7787065074100792	0.2796044949651574
0.7529350386412137	0.7783252279206305	0.2797284613219124
0.4178096124637493	0.7780088444756501	0.2807962966118468
0.4214863357118322	0.2716471092247901	0.2792520883656552
-0.2403075471591297	0.2744725561929851	0.2814502389293977
0.1023318467242938	0.9706470019526096	0.3650082455140010
0.4042308869557770	0.9676816432210917	0.3661086492535659
0.4175871872934908	0.4391777852094442	0.3686457903622855
0.7516750674962590	0.4571167752258907	0.3670930145265879
-0.2404691052717120	0.9577658709085750	0.3686316005647510
0.0845993173488818	0.4348730539429461	0.3665603436490082
-0.0750615075186740	0.6916618059395763	0.3823027249189197
0.5800407371144827	0.6925324976642021	0.3831547109079332
0.5948986497012249	0.1825126140964565	0.3838722436047725
0.2443247434482783	0.6905082876737951	0.3937341067494884
-0.0651979884748656	0.1938072155579237	0.3917097554337889
0.2508173502695366	0.1747096359481970	0.3904962381822774

Optimized XYZ position for the transition states of CH-O breakage on ZnO BCT surface with 0.33 ML OVs

```
AutoCreatByScript
  1.00000000000000
    9.7516200000000008    0.0000000000000000    0.0000000000000000
    0.0000000000000000   10.4606600000000007    0.0000000000000000
    0.0000000000000000    0.0000000000000000   24.3835499999999996
  H    C    O    Zn
    1     1    47    48
Direct
  0.3085120449679865  0.5078617037754100  0.4558253776090622
  0.2778832483224916  0.5115286084252169  0.4125696726597224
  0.2534539999999978  0.2088630000000009  0.0123030000000028
  0.5867870000000012  0.2088630000000009  0.0123030000000028
 -0.0798800000000027  0.2088630000000009  0.0123030000000028
  0.2534539999999978  0.7088630000000009  0.0123030000000028
  0.5867870000000012  0.7088630000000009  0.0123030000000028
 -0.0798800000000027  0.7088630000000009  0.0123030000000028
  0.0867859999999965  0.4588630000000009  0.0507869999999997
  0.4201199999999972  0.4588630000000009  0.0507869999999997
  0.7534530000000004  0.4588630000000009  0.0507869999999997
  0.0867859999999965  0.9588630000000008  0.0507869999999997
  0.4201199999999972  0.9588630000000008  0.0507869999999997
  0.7534530000000004  0.9588630000000008  0.0507869999999997
  0.4220453256420054  0.2121736443563730  0.1292653612456670
  0.0865763481891842  0.2123491515329607  0.1290506347115397
  0.7527852841922801  0.2127735489292150  0.1295453442889848
  0.7537564121864496  0.7120984529709634  0.1295991071670134
```

0.0869713539213038	0.7130197507640186	0.1295840617977740
0.4224943981410610	0.7125075846342072	0.1294423972690906
0.2554166154529760	0.4643998883659591	0.1661041298950167
-0.0805173790557452	0.9624777584680320	0.1658185726685542
0.2543891494926049	0.9623460039484104	0.1654191069566840
0.5879940579657021	0.9624204293231263	0.1668057667593060
0.5882063919484234	0.4635208554107846	0.1667880521840473
-0.0780106455483273	0.4648567344185123	0.1667799764721684
0.2545931269700251	0.2139289150937523	0.2408489595617012
-0.0793542246143563	0.2138114004470838	0.2421940918851401
0.5875973907136071	0.7125016520666791	0.2450983023307508
-0.0811259614491314	0.7135033998886265	0.2444107703682714
0.2555728410245611	0.7097945101951424	0.2423805675965484
0.5882808351808444	0.2120276536787818	0.2447856847837799
0.0877702311654200	0.9641845442225033	0.2776850834226147
0.7552688163142749	0.9681834373257521	0.2849514689899113
0.4198474205966027	0.9654990351829094	0.2803441773447927
0.7564889550074527	0.4638396386110381	0.2812956842087974
0.4235018158368462	0.4593993232006768	0.2821118805136907
0.0906809188424319	0.4680747704412533	0.2841233410133123
0.0972738309200639	0.2361170544012518	0.3534980817805487
0.4126159728805320	0.7701674207368860	0.3622671155203177
0.7507523383609583	0.7672852365781647	0.3632037994854042
0.0876230587178616	0.7776809034380626	0.3600127749981062
0.7602496668392327	0.2631869676840726	0.3631303659027799
0.4204826282463033	0.2433725255520599	0.3617076263883561
0.5862676471763135	0.0153203211290917	0.4040563275112268
-0.0797053961769316	0.5187650053107028	0.3966263191716252
0.5916528376922251	0.5117952836370798	0.3954626492211784

-0.0781956320805549	0.0189309975532137	0.4035237109073095
0.1493269662807198	0.3397591254531755	0.4536211332460524
0.2534539999999978	0.0187629999999999	0.0123030000000028
0.5867879999999985	0.0187629999999999	0.0123030000000028
-0.0798789999999983	0.0187629999999999	0.0123030000000028
0.2534539999999978	0.5187629999999999	0.0123030000000028
0.5867879999999985	0.5187629999999999	0.0123030000000028
-0.0798789999999983	0.5187629999999999	0.0123030000000028
0.0867859999999965	0.2687629999999999	0.0507869999999997
0.4201189999999997	0.2687629999999999	0.0507869999999997
0.7534530000000004	0.2687629999999999	0.0507869999999997
0.0867859999999965	0.7687629999999999	0.0507869999999997
0.4201189999999997	0.7687629999999999	0.0507869999999997
0.7534530000000004	0.7687629999999999	0.0507869999999997
0.4216367735761483	0.0242670036316583	0.1279312000238886
0.0866366325341153	0.0242657685301710	0.1279176235743392
0.7533800764773515	0.0247934990420467	0.1276664838548092
0.0881982211330383	0.5245733880961281	0.1283800099648720
0.4222778918896820	0.5240972478601749	0.1282762910583430
0.7550892121857382	0.5235846742373887	0.1285513556248130
0.2541649647702516	0.2754098377564285	0.1639569790390800
-0.0798760353414502	0.2756051137580670	0.1650276794050384
0.2550423672943006	0.7727492954275386	0.1646080128861822
0.5881933764718931	0.7723398001615729	0.1663722536934001
0.5868962371736637	0.2739954191645969	0.1665990153627143
-0.0801639819169796	0.7724527122363798	0.1659322893099524
-0.0833070947988978	0.0262272539067242	0.2420154878434015
0.2539368896849490	0.0274554223140319	0.2415124000518944
0.5920924164670668	0.0238506735566193	0.2433310903957886

0.5894848865078601	0.5209321639288469	0.2447901208908770
0.2544102765411492	0.5180299907812281	0.2451221856945376
-0.0738902884998102	0.5215625531960872	0.2458146025660065
0.0888930827174773	0.2814414212356828	0.2770347348586968
0.4179768391556608	0.7722550349819788	0.2776684835357363
0.0900218240915161	0.7719989228527917	0.2757933502684688
0.7536880715089305	0.7783381552608221	0.2795262839053348
0.7568808523216342	0.2751145959199569	0.2787733929853680
0.4175118798154083	0.2690413129084361	0.2791526261074616
0.4531556802972215	0.9534374948026754	0.3574995525842137
0.4244838540978250	0.4502513569804167	0.3640192743933582
0.7554639421425552	0.9563036135575754	0.3713157994019063
0.0524740234191286	0.9635471280061749	0.3542743223271326
0.7585282611295181	0.4552293829755413	0.3644737303925462
0.0892149413247387	0.4375155859695795	0.3885877419748262
-0.0766931962511294	0.6894452107226698	0.3810781049211705
0.2413862197823502	0.6836545400767352	0.3874879302546706
0.5812628363755132	0.6850271385899177	0.3832840635101926
0.5946584841965001	0.1899862155452187	0.3867148062759735
-0.0684389679821196	0.1968883760282583	0.3893525447471210
0.2579211406201035	0.2543855155743776	0.4035255396914567

5. References

[1] Mahoney, M. W.; Drineas, P., CUR Matrix Decompositions for Improved Data Analysis. *PNAS* **2009,** *106*, 697–702.

[2] Bernstein, N.; Csányi, G.; Deringer, V. L., De novo exploration and self-guided learning of potential-energy surfaces. *npj Comput. Mater.* **2019,** *5*, 1-9.

[3] Liu, X.; Zhou, W.; Yang, Y.; Cheng, K.; Kang, J.; Zhang, L.; Zhang, G.; Min, X.; Zhang, Q.; Wang, Y., Design of efficient bifunctional catalysts for direct conversion of syngas into lower olefins via methanol/dimethyl ether intermediates. *Chem. Sci.* **2018,** *9*, 4708-4718.

[4] Chen, S.; Ma, S.; Liu, Z.-P., Zirconia-supported ZnO single layer for syngas conversion revealed from machine-learning atomic simulation. *J. Phys. Chem. Lett.* **2021,** *12*, 3328-3334.

[5] Jiao, F.; Li, J.; Pan, X.; Xiao, J.; Li, H.; Ma, H.; Wei, M.; Pan, Y.; Zhou, Z.; Li, M., Selective conversion of syngas to light olefins. *Science* **2016,** *351*, 1065-1068.
